# Using Solid-State NMR to Understand the Structure of Plant Cellulose

**DOI:** 10.1021/jacs.5c14452

**Published:** 2025-12-11

**Authors:** Rosalie Cresswell, Parveen Kumar Deralia, Yoshihisa Yoshimi, Tomohiro Kuga, Alberto Echevarría-Poza, W. Trent Franks, Steven P. Brown, Ray Dupree, Paul Dupree

**Affiliations:** † Department of Physics, 2707 University of Warwick, Coventry CV4 7AL, U.K.; ‡ Department of Biochemistry, 2152 University of Cambridge, Hopkins Building, The Downing Site, Tennis Court Road, Cambridge CB2 1QW, U.K.; § Department of Biomaterial Sciences, Graduate School of Agricultural Life Sciences, University of Tokyo, 1-1-1 Yayoi, Bunkyo-Ku 113-8657, Japan

## Abstract

The structure of plant cellulose microfibrils remains elusive, despite the abundance of cellulose and its utility in industry. Using 2D solid-state NMR of ^13^C-labeled never-dried plants, six major glucose environments are resolved, which are common to the cellulose of softwood, hardwood, and grasses. These environments are maintained in isolated holocellulose nanofibrils, allowing more detailed microfibril characterization. We show that there are only two glucose environments that reside within the microfibril core. These have the same NMR ^13^C chemical shifts as tunicate cellulose Iβ center and origin chains, with no cellulose Iα being detected. The third major glucose site within spectral domain 1, previously assigned to the crystalline microfibril interior, is in close proximity to water, which could indicate that it is a surface glucose environment. The NMR peak widths of all four surface glucose environments are similar to those of the core, indicating that their glucose local order is comparable; there is no significant “amorphous” cellulose in the microfibrils. Consequently, the ratio of the carbon 4 peaks at ∼89 and ∼84 ppm, which has often provided a sample cellulose crystallinity index, is not a meaningful measure of fibril crystallinity or the interior to surface ratio. The revised ratio for poplar wood microfibrils is estimated to be 1:2, which is consistent with an 18-chain microfibril having 6 core and 12 surface chains, although other microfibril sizes are possible. These advances substantially change both the interpretation of solid-state NMR studies of cellulose and the understanding of cellulose microfibril structure and crystallinity.

## Graphical abstract

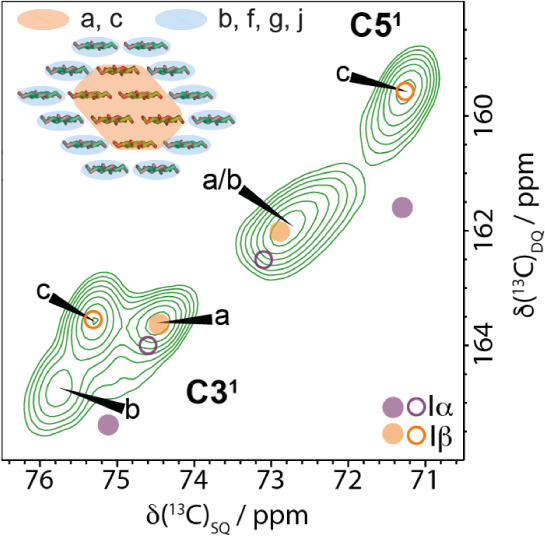

## 1 Introduction

Cellulose is the most abundant natural polymer in the world.^1^ It forms a major component in the cell walls of plants, providing much of their mechanical strength.^2^ Its properties are important for the pulp and lumber industries, and more recently, cellulose has become a major contender as a sustainable alternative to fossil fuels for producing materials, films, and biofuels.^3–5^ Despite the large industrial interest in cellulose, its structure in native plant cell walls remains elusive.

Cellulose is a β-1–4-linked polymer of d-glucose in long chains of perhaps 10,000 units.^6^ These glucan chains have a 2-fold helical structure, which allows them to crystallize via both stacking interactions and a network of hydrogen bonding to form long microfibrils in plant cell walls.^7^ Estimates of the diameter of these cellulose microfibrils are between 3 and 5 nm.^8^ In recent work, based on biophysical measurements and advances in understanding the biosynthesis, it is believed that a microfibril is formed from 18 to 24 glucan chains.^9–15^ The arrangement of these chains in the microfibril into several stacked sheets also remains unknown, but computational studies have suggested some possible habits.^8,16–18^

Cellulose Iα and Iβ are well-known forms of crystalline cellulose and have been characterized using both diffraction techniques and solid-state NMR.^19–23^ Cellulose Iα has a P1 triclinic structure and has nonequivalent glucose residues in a cellobiose unit within the same chain, whereas cellulose Iβ has a P2_1_ monoclinic structure and its two nonequivalent glucose residues are in alternating sheets of glucan chains, called center and origin.^19,20^ It is known that cellulose from bacteria and some algae has predominantly a cellulose Iα structure, while tunicate cellulose, which consists of large crystals, is cellulose Iβ. Although it is possible to obtain high-resolution X-ray and neutron diffraction patterns for these larger crystalline forms of cellulose, this is not achievable for native plant cellulose.^24–26^ The very thin plant microfibrils result in low-resolution diffraction patterns such that atomic resolution cannot be achieved.^26–28^

Solid-state magic angle spinning (MAS) NMR has been used for studying cellulose since the 1980s, mainly using 1D ^13^C cross-polarization (CP) experiments.^29–31^ It is particularly useful as, in contrast to diffraction, it does not require long-range order, and samples can be analyzed in situ. Nevertheless, the thin nature of the plant microfibrils means that the surface glucose residues contribute to the NMR spectra far more than in large cellulose crystals of tunicates or fibrils of bacteria, partially obscuring the signals of the microfibril core and complicating attempts to fully assign the spectra. Furthermore, spectra of whole plant samples contain noncellulosic spectral peaks, as well as some broader components potentially arising from a so-called amorphous cellulose component, that fall in the same region as those of microfibrillar cellulose.^32,33^ The cellulose microfibrils also interact with other components in the cell wall, which is likely to influence the ^13^C chemical shifts of surface glucose residues.^34–36^ Within these limitations, it is widely thought, based on 1D NMR assignments and the fitting of X-ray diffraction patterns, that cellulose of higher plants is a mixture of mainly cellulose Iβ and some cellulose Iα.^28^ The poor match to the ^13^C NMR chemical shifts of these two cellulose I allomorphs has also led several studies to suggest that plant cellulose could have its own distinct structure.^37,38^

In NMR spectra of cellulose, the carbon chemical shifts of C4 and C6 of the different glucose units in cellulose are split into two main regions which, following Dupree,^35^ are defined as spectral domain 1 glucosyl residues, which have C4^1^ and C6^1 13^C chemical shifts of ∼89 ppm and ∼65 ppm, respectively, and spectral domain 2 glucosyl residues, which have C4^2^ and C6^2 13^C chemical shifts of ∼84 ppm and ∼62 ppm. Glucose residues with ^13^C chemical shifts in spectral domain 1 have been classed, largely by groups studying cellulosic materials such as pulp, to reflect crystalline cellulose, as their shifts are close to those of the crystalline cellulose Iα and Iβ,^32,39^ while spectral domain 2 is then in turn assigned as arising from amorphous cellulose. This leads to the ratio of signals in these two domains frequently being defined as the crystallinity index (CI) of a sample and is used alongside crystallinity measures from XRD, despite large differences in their values.^17,40,41^ In other research groups, domain 1 glucose residues are considered to be interior chains of the microfibril^31,32,42^ and domain 2 glucose residues are believed to be surface glucan chains of the cellulose microfibril.^31,32,39^ Using this alternative assignment has led to the ratio of the two domains being used to provide an estimate of the size of the microfibril and has reignited the debate of how many chains form a microfibril.^9,11,15^ When restricted to 1D ^13^C MAS NMR, it is only possible to resolve residues in these two domains using C4 and C6 shifts, as the C5 shifts overlap with those from C2 and C3, and all the C1 shifts of all glucose residues also overlap.^35^ The C4 cellulose region of the 1D ^13^C NMR spectrum tends to be reasonably clear of contributions from hemicelluloses and pectin, and so the ratio of the C4 carbons of the two domains (C4^1^:C4^2^) has been widely used to characterize and compare the cellulose in different plants and cellulosic materials.^43,44^ It has been suggested^45^ that the major source of this change in ^13^C chemical shift between the two domains seen in carbons 4, 5, and 6 arises from a change in the conformation of the hydroxymethyl group of the glucose residue from *tg* in spectral domain 1 to *gt* or *gg* in spectral domain 2, and this has since been supported by several DFT and NMR studies.^18,46,47^

In recent years, the availability of high-field NMR spectrometers and developments in ^13^C labeling of plant cell wall materials (and dynamic nuclear polarization (DNP)) have enabled access to a wealth of 2D MAS NMR experiments that have allowed unparalleled insight into the structure of plant cell walls and other biological polymer matrices.^48–50^ These studies have made significant strides in understanding hemicellulose structure and interactions with cellulose in both primary (PCW) and secondary cell walls (SCW).^51,52^ Solid-state NMR has been used to identify the 2-fold helical structure of xylan in SCW, and the molecular architecture and water–polymer interactions in softwoods.^34,52,53^ With an aim of understanding the structure of the plant cellulose microfibrils, several distinct glucosyl residues, each with differing ^13^C chemical shifts due to their different local environments, were described by Wang et al., who found similar glucose environments in several plants (*Brachypodium*, *Zea mays*, and *Arabidopsis thaliana*).^43,54^ They suggested cellulose microfibril models based on their understanding that the five domain 1 glucose environments arise solely from sites in the interior chains of the microfibril and the two domain 2 environments lie on the surface.^47,55^ Under this assumption, and using quantitative NMR, they found that cellulose had too many interior relative to surface residues for the favored microfibril size of 18–24 chains. Additionally, some of the “interior” residues were more distant from water than others, which is difficult to reconcile with an 18–24 chain fibril. These discrepancies between the fibril models and NMR data led to the suggestion that the cellulose microfibrils bundle, producing a cellulose structure with two layers of interior glucose residues with differing water proximity.^43^

In this work, we assign the ^13^C NMR chemical shifts of the main cellulosic glucose environments found in a range of plant secondary cell wall materials and begin to determine their positions in the cellulose microfibril. To achieve this, we studied both poplar wood and xylanase-treated holocellulose nanofibrils (hCNFs) prepared from poplar wood using minimal processing to maintain the native cellulose structure.^56^ By removing hemicellulose, lignin, and any amorphous cellulose present, we are left with a clearer spectrum with improved resolution, which provides more certainty in the assignment of ^13^C NMR chemical shifts for each distinct glucose environment. By comparing with the recently corrected ^13^C NMR chemical shift assignments for cellulose I^23,57,58^ and our spectra of tunicate cellulose, we conclusively show that plant cellulose does not contain the Iα polymorph and that the core of native cellulose microfibrils from diverse plant sources has ^13^C NMR chemical shifts identical to the tunicate cellulose Iβ polymorph. We also show that categorizing the glucose residues by their chemical shifts into spectral domain 1 or domain 2 does not reflect a split into crystalline and amorphous cellulose nor does it exclusively reflect interior and surface cellulose. In particular, we find that one of the domain 1 glucosyl residue environments is close to water, indicating that it could be part of a surface glucan chain. (To assist the reader, Tables S4 and S5 list key terms and acronyms used in this work.) Taken together, these findings identify and resolve several widely held misconceptions and misinterpretations of solid-state NMR data and provide a strong basis to build more coherent models for cellulose in plant cell walls.

## 2 Results

### 2.1 Assigning ^13^C NMR Chemical Shifts for the Glucosyl Residue Environments in Cellulose within Plant Cell Walls

Throughout our work,^34,53,59,60^ never-dried samples of many different plants have been studied using ^13^C MAS NMR to understand the structure and interactions of cellulose, hemicellulose, lignin, and water in native plant cell walls. The secondary cell wall of poplar wood is relatively simple, with only 3 main components: cellulose, xylan, and lignin. Figure 1 shows that the 1D cross-polarization (CP) MAS NMR spectrum of the neutral carbohydrate region is dominated by the cellulose signal. The carbon 1 (C1) peak is at ∼105 ppm; C2, C3, and C5 overlap in the region between 70 and 76 ppm, and the C4 and C6 carbons are split into two main peaks. The cellulose glucose environments in spectral domain 1 show C4^1^ and C6^1^ peaks at ∼89 ppm and ∼65 ppm, respectively, and the C4^2^ and C6^2^ spectral domain 2 peaks are at ∼84 ppm and ∼62 ppm, respectively. Both these C4 and C6 regions in the 1D CP MAS spectrum are composed of several peaks since multiple glucose environments contribute to these spectral domains. The C4^2^ region especially is clearly split into two peaks at ∼83.5 and ∼84.5 ppm.

**Figure 1:**
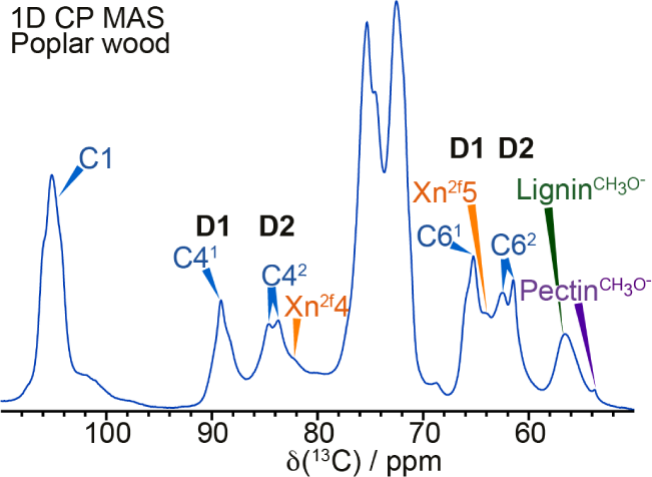
The neutral carbohydrate region of a ^13^C 1D CP MAS NMR spectrum of poplar wood. The spectrum shows the assignments of cellulose (blue), xylan (orange), lignin (green), and pectin (purple). The glucose C4 and C6 peaks in cellulose are split into two regions named spectral domain 1 (D1) and spectral domain 2 (D2). The spectrum was recorded at a ^13^C Larmor frequency of 251.4 MHz and a MAS frequency of 12.5 kHz.

Fully ^13^C-labeled never-dried poplar wood enables the structure of plant cellulose to be explored using a wealth of 2D MAS NMR experiments.^34,43,61,62^ The CP MAS refocused INADEQUATE NMR experiment^63,64^ is a double-quantum experiment that correlates two covalently bonded carbons, with directly bonded carbons appearing at the same Double Quantum (DQ) shift, given by the sum of the respective Single Quantum (SQ) shift of the bonded carbons. Figure 2 shows the neutral carbohydrate region of a ^13^C CP MAS refocused INADEQUATE NMR spectrum of poplar wood. The high resolution means that the carbons within each glucosyl residue can be followed through in the 2D spectrum.

**Figure 2:**
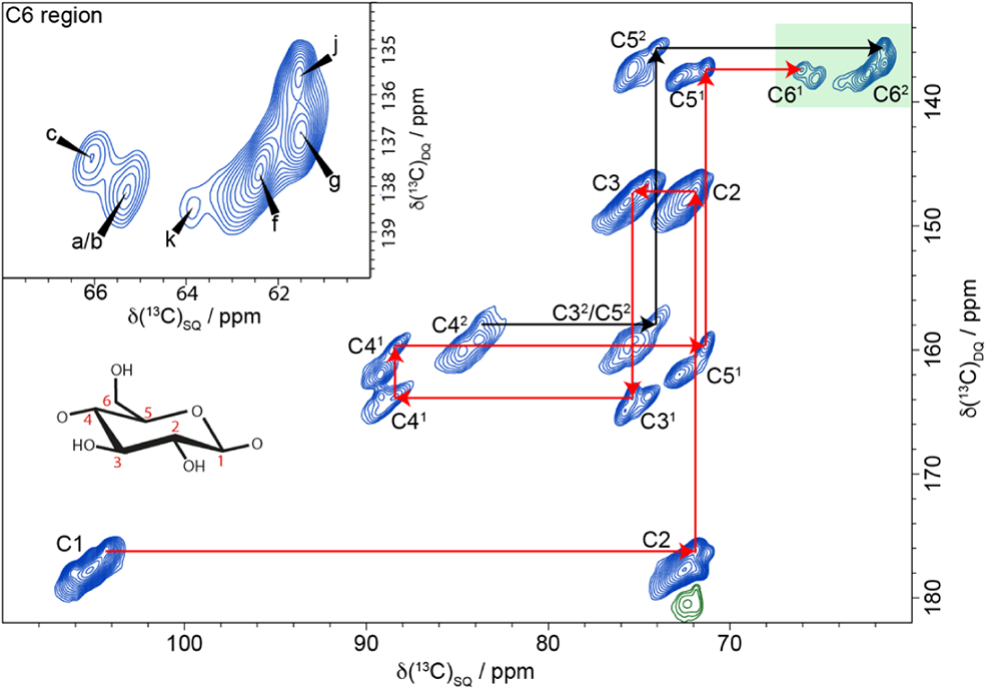
A ^13^C 2D CP refocused INADEQUATE MAS NMR spectrum of never-dried poplar wood showing multiple glucose environments in both spectral domains 1 and 2. The path of ^13^C carbon chemical shifts for cellulose glucose residue environments c (red) and j (black) is shown as example assignments. Inset (top left): The C6 region with the six major glucose environments identified in poplar wood (a, b, c, f, g, j). The spectrum was recorded as in Figure 1.

This is demonstrated in red for a glucose residue in domain 1 named environment “c” and in black for the C4–C6 region for a residue in domain 2 environment “j”. Our naming convention and assignments are based on those of Wang et al.^54^ for cellulose in primary cell walls, since many of the cellulose environments we observe have similar values. However, glucose environment j, which is clearly visible for C4, C5, and C6 in the ^13^C CP MAS refocused INADEQUATE NMR spectrum, is newly reported here. The C6 region of the spectrum (inset in Figure 2) shows the main glucose environments identified within spectral domain 1 and spectral domain 2. This region also shows a minor domain 2 environment, named k. We found, in contrast to previous work on plant primary cell walls,^54^ that there are just six major glucose environments in this never-dried poplar sample: three (a, b, c) in spectral domain 1 and three (f, g, j) in spectral domain 2.

To make a more complete assignment of the ^13^C NMR chemical shifts of the glucose environments within cellulose, a 30 ms ^13^C CP MAS proton-driven spin-diffusion (PDSD) NMR spectrum was analyzed alongside the ^13^C CP MAS refocused INADEQUATE NMR spectrum. The ^13^C CP MAS PDSD NMR experiment is a through-space correlation experiment for which, with a mixing time of 30 ms, the observed cross-peaks are found to only correlate carbons that are within the same glucose ring (i.e., within ∼3–4 Å). The 30 ms CP MAS PDSD NMR spectrum shown in Figure S1 highlights the key regions (C6–C1, C6–C4, C6–C5, and C4–C1), which are particularly useful for identifying different glucose environments. Two further minor glucose environments in domain 1, labeled d and e (which are very minor in our poplar wood sample) have previously been assigned by Wang et al.^54^ Table 1 lists the ^13^C NMR chemical shifts for the six major and the minor glucose environments identified in cellulose of poplar wood.

**Table 1:** NMR Chemical Shifts of All Glucose Environments Assigned in the Cellulose of Poplar Wood

**Spectral Domain 1**						
**Glucose Environment**	**C1** ^**1**^	**C2** ^**1**^	**C3** ^**1**^	**C4** ^**1**^	**C5** ^**1**^	**C6** ^**1**^
a* (Cellulose Iβ origin)	106.1^†^	71.8	74.5	89.2	72.8	65.2
b	105.4	72.6	75.8	89.3	72.7	65.4
c (Cellulose Iβ center)	104.3	71.8	75.4	88.4	71.3	66.0
*d*	*105.2*	*72.5*	*74.9*	*87.1*	*72.5*	*64.7*
*e*	*105.0*	-	*74.7*	*89.8*	*71.1*	*65.3*

**Spectral Domain 2**						
**Glucose Environment**	**C1** ^**2**^	**C2** ^**2**^	**C3** ^**2**^	**C4** ^**2**^	**C5** ^**2**^	**C6** ^**2**^
f	105.2	72.4	74.4	84.5	75.3	62.4
g	105.0	72.4	75.3	83.5	75.2	61.5
j	105.1	-	-	83.3	73.9	61.4
*k*	-	-	-	*83.8*	*74.3*	*63.7*

*The naming convention and assignments are based on those of Wang et al.,^54^ with additional environments such as j assigned in this work. The minor glucose environments d, e, and k are italicized.

^†^All ^13^C NMR chemical shifts are in ppm with an error of ±0.1 ppm.

### 2.2 Glucose Environments in Cellulose in Cell Walls of Different Plants

Having identified the six glucose environments in the cellulose of poplar wood, we were interested in the presence of these in the cellulose of other plant species. Over the years, we have used high-resolution 2D MAS NMR experiments to study a wide range of different plants, including monocots, eudicots, and gymnosperms.^52,59,60^ The spectra have strikingly similar cellulose NMR shifts, such that the six major glucose environments a, b, c, f, g, and j can be seen in a wide range of plants. This is illustrated in Figure 3 using a comparison of the 30 ms ^13^C CP MAS PDSD NMR spectra of a eudicot (poplar), a monocot grass (*Brachypodium*), and a gymnosperm (spruce) in the C4–C1 and the C6–C4 regions. These six major glucose environments identified in poplar are always present in the other plants, but the relative amounts of each environment vary. For example, spruce wood appears to have relatively more of site b versus a or c, whereas in poplar wood cellulose, these are similar in quantity. Regarding the minor glucose environments, *Brachypodium* has significantly more of site d compared to both poplar and spruce wood. The environment e as observed in primary cell walls,^54^ while not visible in Figure 3, is very minor in all three plants. There is one additional environment in domain 1 of the spectra of spruce, which we speculate could arise due to a different microfibril habit or cellulose interactions with galactoglucomannan, the major hemicellulose in softwoods. There are some small differences in the shifts for some of the minor glucose environments between plants. For example, we found that the minor environment d is generally broader than sites a, b, and c, with its C4 ^13^C NMR chemical shift varying by ∼0.3 ppm, indicating that there are some differences in the local environment of site d. In summary, the six major glucose environments (a, b, c, f, g, and j) can be seen in the cellulose microfibrils of all the plants we have studied, reflecting common aspects of the structure of the cellulose microfibrils. The differences in abundance of the glucose environments could arise from changes in microfibril size or shape, as well as interactions with hemicelluloses and from different proportions of fibril types in different plant tissues. Therefore, identifying the location of each of these glucose environments within the microfibril will provide invaluable insight into the different cellulose structures observed across the various plant cell walls.

**Figure 3:**
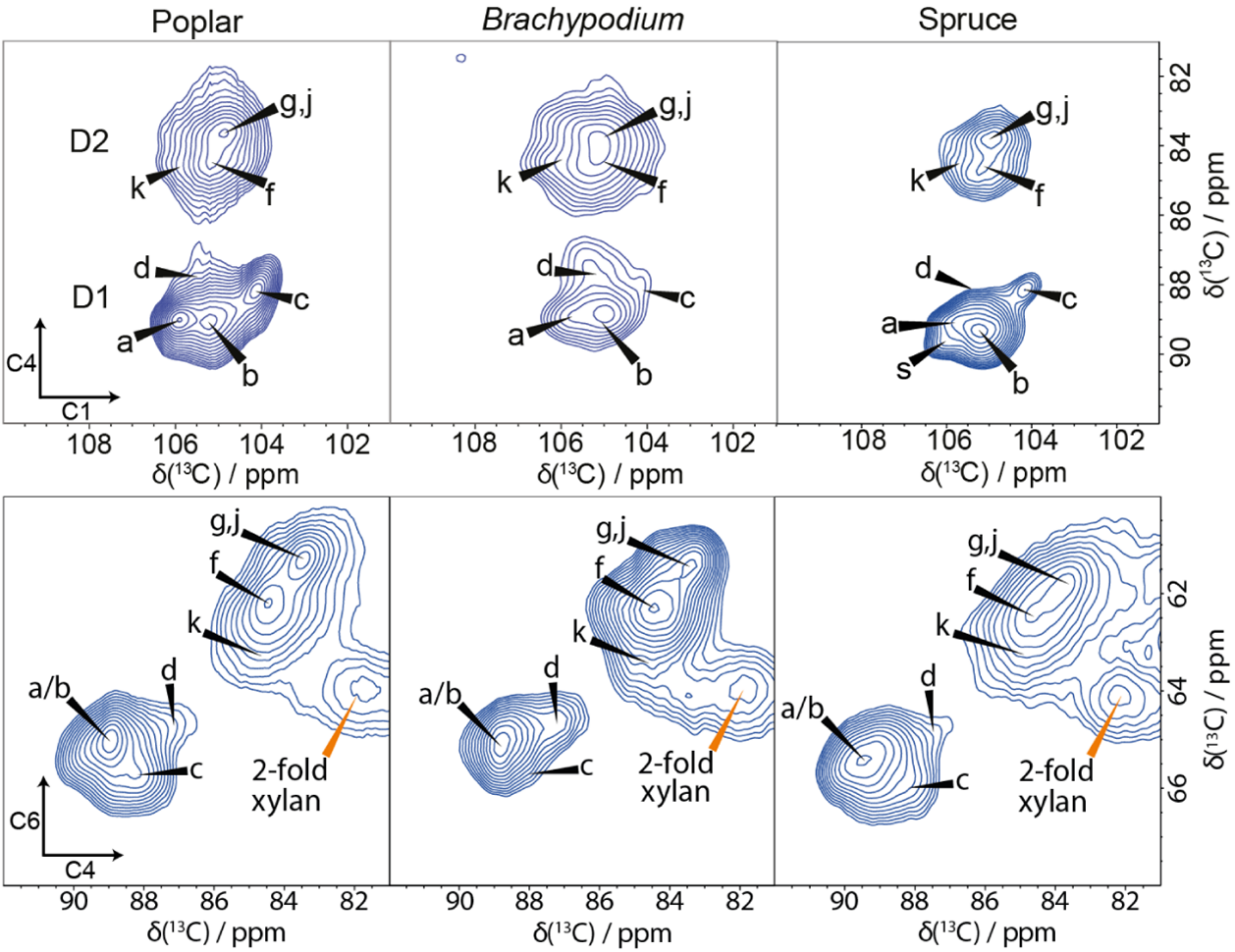
Glucose environments in cellulose are common across different plants. A comparison of the C4–C1 and C6–C4 regions of 30 ms ^13^C 2D CP MAS PDSD NMR spectra of poplar wood (a eudicot), *Brachypodium* mature leaves (a monocot), and spruce wood (a gymnosperm). Common glucose environments are labeled. Note that, while the relative amounts vary, the cellulose environments are common throughout. The spectra were recorded as in Figure 1.

### 2.3 Xylanase-Treated Holocellulose Nanofibrils (hCNFs) Maintain the Native Plant Cellulose Structure of Poplar Wood

While studying the native plant cellulose in situ is ideal to ensure minimal disruption, the ^13^C MAS NMR spectrum can be crowded since signals from hemicelluloses and lignin are present within the same region, which tends to complicate the analysis and limit the resolution of cellulose environments in the spectrum. The glucose environments in the microfibrils may also be influenced by interactions of surface residues with hemicellulose or lignin. Therefore, we delignified the poplar wood using peracetic acid (PAA) to oxidize and remove the lignin while maintaining the cellulose microfibril structure. It has been shown that this method does not substantially affect the cellulose throughout this process.^65^ By removing the lignin during preparation of holocellulose nanofibrils (hCNFs),^56^ we can study the cellulose microfibril structure while simplifying the ^13^C MAS NMR spectrum. TEM images, seen in Figure S2, of the hCNFs sample show long and thin, loosely bundled cellulose microfibrils. The width of the fibrils was measured across many points over several TEM images of the sample, giving an estimate of ∼3 nm in width for the xylanase-treated hCNFs of the poplar wood. To reduce any effect on the spectrum of the xylan presence, we removed the majority of xylan using xylanase hydrolysis. We compared the ^13^C MAS NMR spectra of poplar wood to those of the xylanase-treated hCNFs to ensure the major cellulose environments are maintained. The comparison of the 1D ^13^C CP MAS NMR spectra is shown in Figure 4a. There is a distinct increase in the apparent resolution of the 1D spectrum for the hCNFs sample, which is particularly evident in the C1 region and to a lesser extent in the C6 region. There is also a significant change in the total signal in the C4 region as well as the ratio of the domain 1 and domain 2 cellulose peaks. This change is partly due to the removal of both lignin and hemicelluloses as well as, perhaps, the loss of a less ordered (broad) cellulose component that is not part of the cellulose microfibril. This more disordered component is removed during the production of the hCNFs, while the xylanase treatment of the hCNFs sample removes most of the xylan hemicellulose without affecting the ratio of the two cellulose domains, as shown in Figure S3.

**Figure 4:**
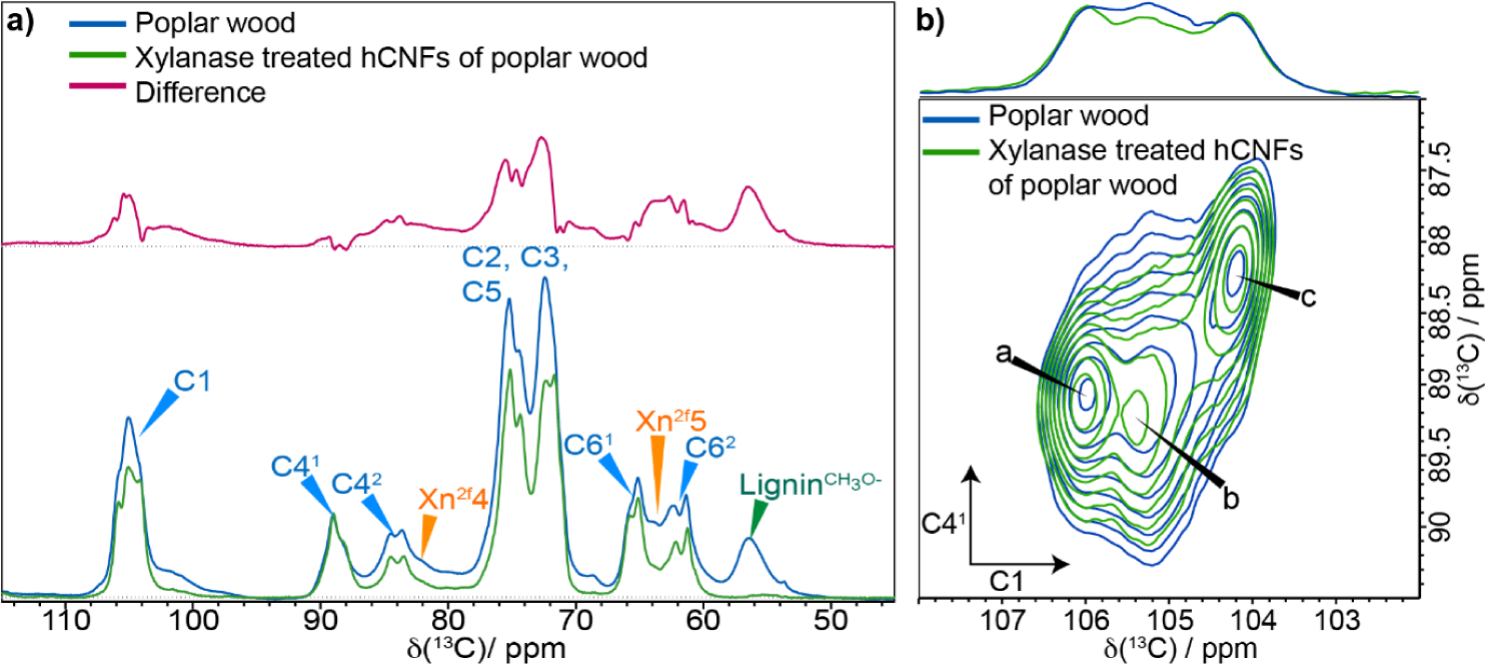
Comparison of poplar wood and xylanase-treated hCNFs of poplar wood. a) The neutral carbohydrate region of the ^13^C 1D CP MAS NMR spectrum. The comparison (normalized to C4^1^ at 89 ppm) shows the difference in the 1D CP spectrum of poplar wood (blue) and xylanase-treated hCNFs (green) when hemicellulose and lignin have been removed. It is clear that the production of hCNFs results in the removal of most of a broad background signal, as shown by the difference (red). b) SCW cellulose of poplar wood maintains its structure in the fibrillation process to form hCNFs. Comparison of the cellulose C4–C1 domain 1 region of 30 ms ^13^C 2D CP PDSD MAS NMR spectra of poplar wood (blue) and xylanase-treated hCNFs of poplar wood (green). Sum projections of the C4–C1 domain 1 region are shown at the top. Spectra were recorded as shown in Figure 1.

A comparison of 2D 30 ms ^13^C CP MAS PDSD NMR spectra of poplar wood and xylanase-treated hCNFs for the C4^1^–C1, C6–C1, and C4^2^–C1 regions, shown in Figures 4b, S4, and S5, respectively, confirms that the domain 1 glucose environments a, b, and c remain almost completely unchanged by the production of hCNFs from poplar wood. The domain 2 environments show some slight ^13^C NMR chemical shift changes, typically <0.3 ppm, in both the f and g/j environments. As the domain 2 environments are surface chains of the microfibril, this could be due to changes in their interactions caused by the removal of both lignin and some xylan. Since there were no substantial changes in the ^13^C NMR chemical shifts, we determined that the production of the hCNFs causes relatively minimal disturbance to the cellulose microfibril structure. Therefore, these isolated fibrils can be used to help identify the six major glucose environments within the native cellulose microfibril. Interestingly, we find that the line widths of the major environments in spectral domain 1 and domain 2 are similar, as illustrated in Figure 5, which shows the C6 region of a CP refocused INADEQUATE spectrum of the xylanase-treated hCNFs. The similar line widths for the major environments, summarized in Table S1, indicate that these different glucose sites in both these spectral domains have a similar local order.

**Figure 5:**
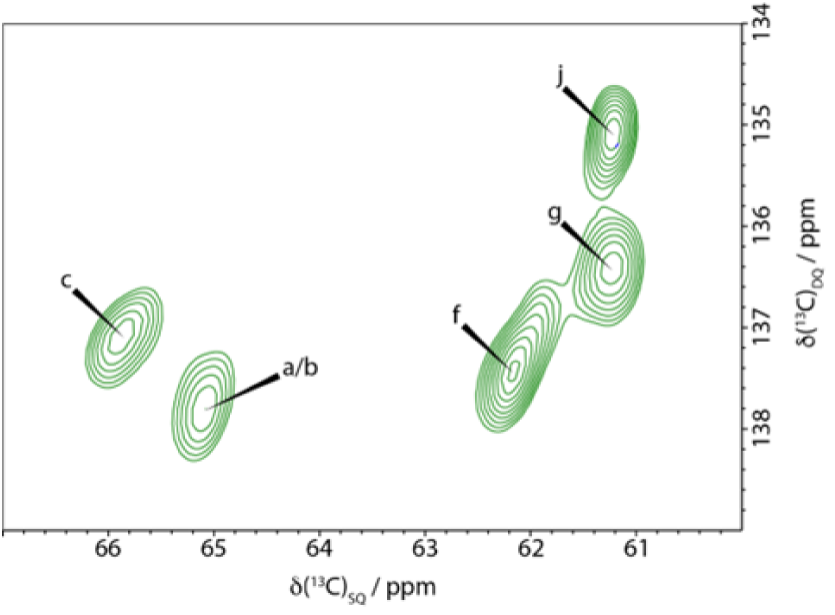
Cellulose fibril surface glucose environments in domain 2 are as well ordered as the glucose environments in domain 1 that include the fibril core. The C6 region of a ^13^C 2D CP refocused INADEQUATE MAS NMR spectrum of xylanase-treated hCNFs of poplar wood. The linewidths of the main domain 2 surface environments (f, g, j) are comparable to those of domain 1 (a, b, c), indicating significant (and similar) local order of the domain 2 glucose residues that lie on the fibril surface. Spectra were recorded as in Figure 1.

### 2.4 Plant Cellulose Microfibril Core Environments Are Identical to Tunicate Cellulose Iβ

The improvement in the resolution of ^13^C MAS NMR spectra makes the analysis of the xylanase-treated hCNFs from poplar wood ideal for assigning the distinct ^13^C NMR chemical shifts to specific locations within the microfibrils. We compared the ^13^C NMR chemical shifts of our xylanase-treated hCNFs to those of a tunicate (cellulose Iβ) sample and to the ^13^C chemical shift assignments of cellulose I by Brouwer and Mikolajewski.^23^ In Figure 6a, a comparison of the 1D CP MAS NMR spectra of tunicate (cellulose Iβ) and the xylanase-treated hCNFs sample shows that the tunicate shifts precisely overlay a subset of the peaks in the hCNFs spectrum. Indeed, the ^13^C chemical shifts for the C1 of a and c match the C1 ^13^C chemical shifts of those for tunicate cellulose. We next compared our ^13^C chemical shift assignments from the high-resolution ^13^C 2D CP MAS refocused INADEQUATE NMR spectrum of xylanase-treated hCNFs with the ^13^C NMR chemical shifts of both cellulose Iα and Iβ. None of the domain 2 surface glucose environments ^13^C NMR chemical shifts are close to those in either of these cellulose allomorphs (Table S2). This means we only need to consider the ^13^C chemical shifts of spectral domain 1 glucose residues, where there are only 3 major sites: a, b, and c.

**Figure 6:**
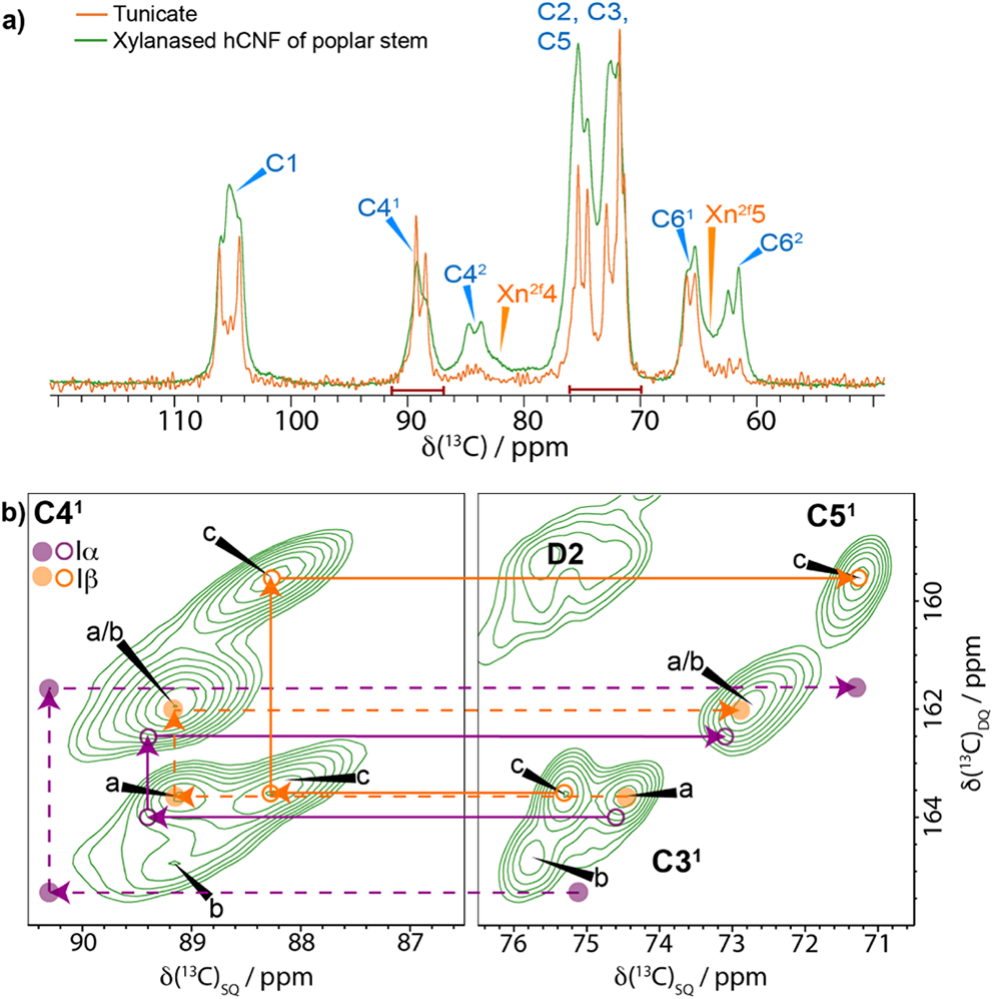
Plant secondary cell wall cellulose has a cellulose Iβ component but no cellulose Iα component. a) Comparison of 1D ^13^C CP MAS NMR spectra of tunicate (orange) and xylanase-treated hCNFs of poplar wood (green). b) Comparison of the domain 1 C4-C3/C5 region of a ^13^C 2D CP refocused INADEQUATE MAS NMR spectrum of xylanase-treated hCNFs of poplar wood with the ^13^C chemical shifts of cellulose Iα and Iβ, as given by Brouwer and Mikolajewski^23,57^ (see Table S2). The filled and empty circles correspond to two distinct ^13^C chemical shifts in the asymmetric unit cells of cellulose Iα and Iβ.^23,57,58^ The Iβ cellulose shifts are within error (±0.1 ppm) identical to those of cellulose sites a and c of xylanase-treated hCNFs of poplar wood. The Iα cellulose positions do not match the ^13^C NMR chemical shifts of any glucose environment acquired from the native never-dried plant cell wall. Spectra were recorded as shown in Figure 1.

Figure 6b shows the domain 1 C4–C3/C5 region of a ^13^C CP MAS refocused INADEQUATE NMR spectrum of the xylanase-treated hCNFs, with the expected Iα and Iβ ^13^C chemical shift positions highlighted on the spectrum. Interestingly, the C3, C4, and C5 shifts of glucose environments a and c, as in the 1D comparison with tunicate, are nearly identical to those of cellulose Iβ. Indeed, all the ^13^C chemical shifts for sites a and c, apart from C1, are within ∼0.1 ppm of those reported for cellulose Iβ (Figure S6 and Table S2).^23^ Since the only substantial difference we observe from the previously reported Iβ ^13^C chemical shifts is in C1, it seems likely that the Kono et al.^21,22^ assignment was incorrect. This is highlighted in Figure S7, where it can be seen that by swapping the C1 assignments, there is alignment with those observed in our 30 ms CP MAS PDSD NMR spectra. This misassignment by Kono et al.^22^ presumably arose because the ^13^C CP MAS refocused INADEQUATE NMR spectrum alone was used for their assignment and both glucose environments in cellulose Iβ have nearly identical C2 ^13^C NMR chemical shifts, making it difficult to distinguish the associated C1 chemical shifts. In contrast, by using the 30 ms CP MAS PDSD NMR spectrum (Figure S7) together with the ^13^C CP MAS refocused INADEQUATE NMR spectrum, we could determine confidently the C1 ^13^C chemical shifts for environments a and c. Indeed, very recently, Brouwer and Mikolajewski have also noted that the Kono et al. C1 assignments should be exchanged.^57,58^

With this new NMR shift assignment of cellulose Iβ, all the shifts of microfibril core glucose environments a and c match closely to those for tunicate cellulose Iβ. Furthermore, we can now assign environment a as the origin chain and environment c as the center chain, since DFT calculations for cellulose Iβ predict the C1 chemical shift of the residues in the origin chains to be ∼2 ppm higher than the C1 of residues in the center chains, as seen here for environments a and c, respectively.^54,66^ We next considered evidence of the presence of cellulose Iα. Despite environment b having similar ^13^C chemical shifts to those of one of the Iα glucose environments for both C1 and C4 (Table S2), there is no sign of the second Iα glucose environment with a C4 at 90.3 ppm, which would be in similar quantities to site b. It is evident from Figure 6 that the shifts of cellulose Iα are very different from those observed for xylanase-treated poplar hCNFs. Since this is true for all the plant samples analyzed here, there is a clear absence of any cellulose Iα in plants. This observation is contrary to the long-held belief that plant cellulose is a mixture composed of largely cellulose Iβ with varying amounts of the cellulose Iα allomorph,^26,30^ whereas, in fact, it is solely a cellulose Iβ structure.

Having determined that two of the main spectral domain 1 environments correspond to center and origin chains in the classical cellulose Iβ structure, we wanted to understand the nature of the third remaining domain 1 environment, b. Since site b is from domain 1, it has been assumed to be interior to the microfibril. However, given that this is not a cellulose Iα or Iβ environment, its position within the microfibril is unclear. To investigate this further, two 2D water-edited NMR experiments were undertaken to probe the glucose environments that are closest to water, specifically (i) a 30 ms CP MAS PDSD and (ii) a refocused CP MAS INADEQUATE experiment. Figure 7a and b shows the C4–C1 and C6–C1 regions of a water-edited PDSD spectrum compared with a standard 30 ms CP MAS PDSD spectrum (see Figure S8 for the full neutral carbohydrate region of the spectrum). The ^1^H *T*_2_ filter used at the start of the experiment to select the magnetization on the water protons alone was optimized for both samples (see Table S3). Figure S9 shows that at short ^1^H *T*_2_ filter times of 0.08 and 0.16 ms, signal remains on the protons of the cellulose as the D1:D2 ratios are significantly different. However, ^1^H *T*_2_ filter times of ≥0.24 ms sufficiently suppress the magnetization of the cellulose protons, leading to identical 1D ^13^C spectra regardless of the ^1^H *T*_2_ filter time. The ^1^H *T*_2_ filter times used were also short enough to ensure a sufficient CP signal for the 2D experiments. As expected for surface glucose residues, the signal for the domain 2 sites f, g, and j is enhanced in both of these regions, indicating that these residues are more water accessible than the microfibril core origin and center chain sites a and c. Interestingly, Figure 7a shows that the water-edited C4–C1 peak for glucose environment b is also significantly enhanced in a similar way to the surface glucan sites in spectral domain 2, indicating that b is also closer to water than the microfibril core sites a and c. This observation suggests that glucose environment b of domain 1 is therefore, unexpectedly, a hydrated environment and potentially part of a surface chain of the microfibril. Interestingly, for the C6–C1 region and C6 region of the CP PDSD and refocused INADEQUATE MAS NMR spectra shown in Figures 7b and S10, respectively, the signal from b is enhanced in the water-edited experiment over the signal from the core a and c environments but to a lesser extent than for the domain 2 surface glucose environments. Being in domain 1, environment b likely reflects a glucose residue with the C6 hydroxymethyl in a *tg* conformation. This conformation may arise because the C6 is facing toward the interior of the cellulose microfibril, where the hydroxymethyl group hydrogen bonds with other glucose residues.^18^ Hence, in the glucose residue of environment b, C6 is further from water than C4 and further from water than the other surface environments, where C6 has a water-facing *gt* or *gg* conformation. The C3/C5 region of the water-edited ^13^C CP MAS refocused INADEQUATE NMR spectrum of both the xylanase-treated hCNFs (Figure 7c) and of poplar wood (Figure 7d) also shows that for both samples, site b is in closer proximity to water than the core glucose environments a and c. This proximity difference is observed for both poplar wood and the hCNFs sample, indicating that it is not an artifact of the hCNFs sample and is representative of the native cellulose microfibril. Additionally, the C6 region of the ^13^C CP MAS refocused INADEQUATE NMR spectrum shows that all three major domain 2 glucose environments are close to water, with perhaps site g being marginally closer compared to sites f and j (Figure S10). In summary, the water proximity experiments reveal that, in contrast to the widely accepted view,^32,43,67^ not all the domain 1 glucose residues are in the core Iβ structure of the cellulose microfibril.

**Figure 7:**
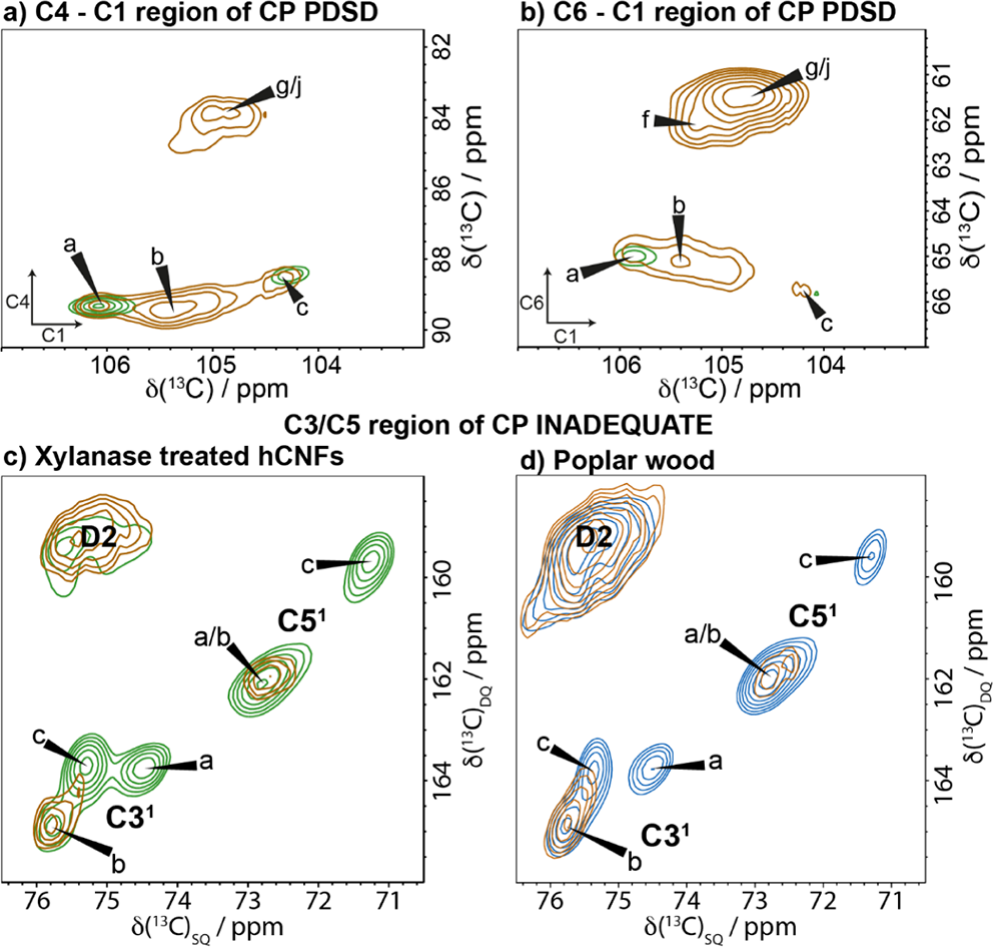
Domain 1 glucose environments in cellulose are not solely interior chains. Comparison of the standard (green) and water-edited (brown) 30 ms ^13^C 2D CP MAS PDSD NMR spectra of xylanase-treated hCNFs of poplar wood. a) is the C4–C1 region, and b) is the C6–C1 region. Spectra were normalized to glucose environment a. c) Comparison of the C3/C5 region of the standard (green) and water-edited (brown) CP refocused INADEQUATE spectra of xylanase-treated poplar wood. d) Comparison of the C3/C5–C4 region of the standard (blue) and water-edited (brown) CP MAS refocused INADEQUATE NMR spectra of poplar wood. Both c) and d) were normalized to glucose environment b. Spectra were recorded as in Figure 1.

To investigate the relative proximities of the different glucose environments within the microfibril, two longer (200 and 400 ms) mixing time ^13^C CP MAS PDSD NMR experiments were performed. As these longer mixing time experiments probe distances up to ∼5–8 Å, additional cross-peaks between glucose residues in adjacent sites within individual fibrils are observed. Figure 8 shows that at a mixing time of 200 ms, the C1–C1 region of a ^13^C CP MAS PDSD NMR spectrum gives clear cross-peaks between a and c only. This shows that the center and origin chain environments a and c are closer to each other than either is on average to residue environment b. This is consistent with our finding that b is a surface environment and is not situated with a and c in the core of the cellulose microfibril. The first clear cross-peaks between glucose residues in the two different domains can be seen in the C4–C4 region of the 400 ms ^13^C CP MAS PDSD NMR spectrum (Figure 9a). There are clear crosspeaks from surface domain 2 glucose environments f and g/j to the single C4^1^ peak corresponding to environments a and b. Figure 9 also shows clear cross-peaks between sites a and b to all D2 environments and weaker cross-peaks from site c to the D2 environments. The cross-peaks across from a and c to the D2 environments indicate that the cellulose Iβ microfibril structure is not in a separate region to the D2 environments, supporting the core shell Iβ model. The larger cross-peaks of a/b to D2 environments indicate that site b is also in close proximity to the D2 surface environments. The proximities of b to the domain 2 environments suggests that b could be one of the four major surface glucose residue environments. There were no cross-peaks between b and a,c at the shorter (200 ms) mixing time (Figure 8), suggesting that b cannot lie within the core with a,c.

**Figure 8:**
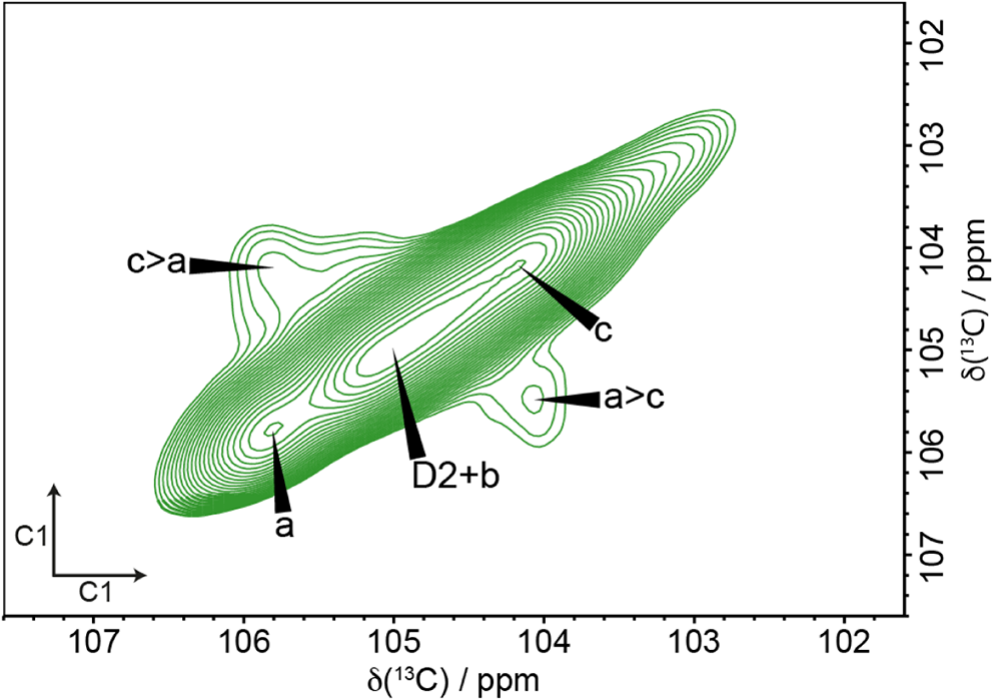
Domain 1 glucose environments a and c are in close proximity. The C1–C1 region of a 200 ms ^13^C 2D CP MAS PDSD NMR spectrum of xylanase-treated hCNFs of poplar wood shows cross-peaks between a and c. Spectra were recorded as in Figure 1.

**Figure 9:**
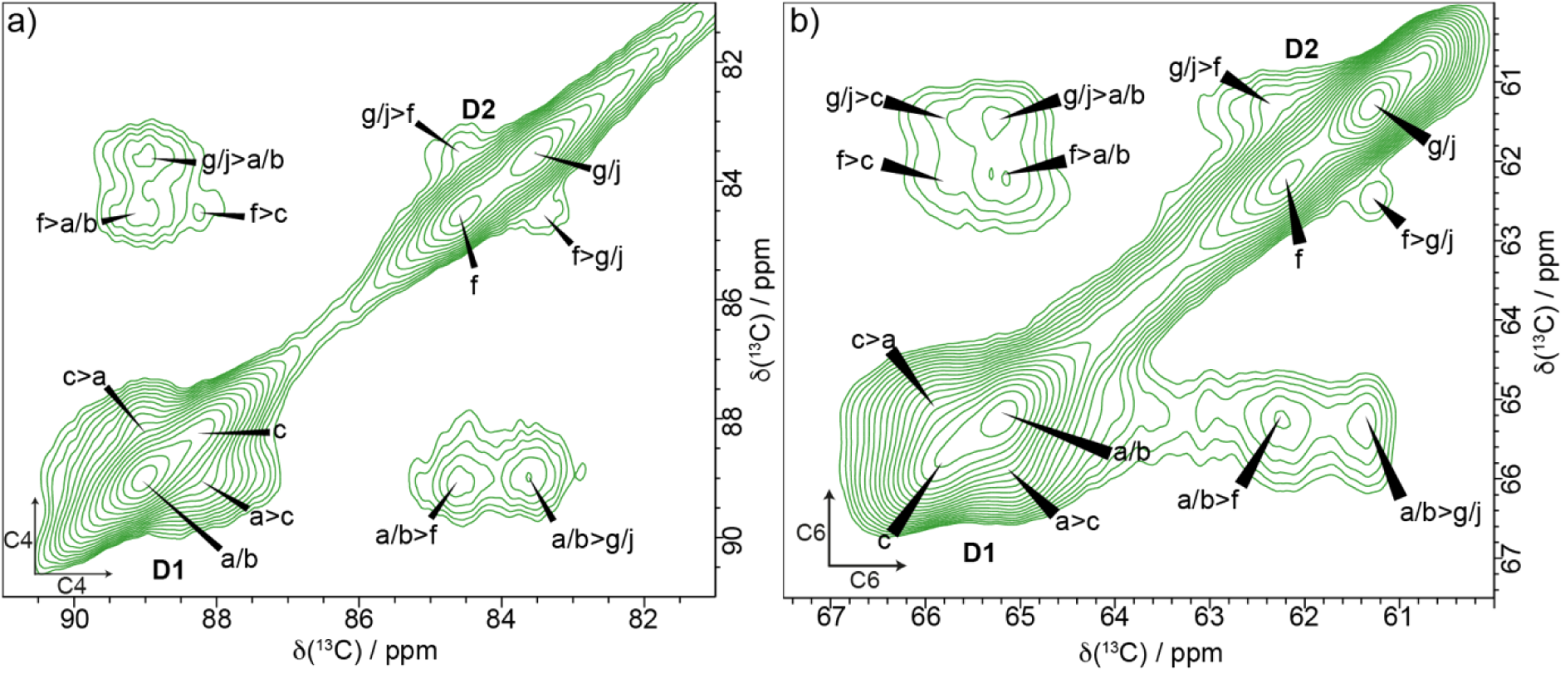
Domain 1 glucose environments and domain 2 glucose environments are in close proximity. A 400 ms ^13^C 2D CP MAS PDSD NMR spectrum of xylanase-treated hCNFs of poplar wood. a) the C4–C4 region, b) the C6–C6 region. Spectra were recorded as in Figure 1.

Given the new assignment of the core (sites a and c) and surface environments (b, f, g, j), the true ratio of interior vs surface of the microfibril can be estimated by integrating the C1 region of the CP refocused INADEQUATE spectrum, where core sites a and c are distinguishable from the surface glucose environments (b + D2) (see Figure S11a). Since the refocused INADEQUATE spectrum is not necessarily quantitative, the reliability of this integration was confirmed by comparing 1D CP, quantitative DP, and 1D double quantum (DQ) filtered spectra for the xylanase-treated poplar hCNFs sample. Two different echo times were used in the 1D DQ filtered experiment to determine whether this affected the relative amplitude of the peaks. When normalized to the core C1 peak (site a) at 106.1 ppm, there was very little change in the relative intensities of different glucose environments within the C1 region, as shown in Figure S11b. Hence, the integration of the C1 area of the CP refocused INADEQUATE (see Figure S11a) can be used to give an estimate of the relative amount of interior sites a and c versus surface sites b + D2 (f, g, j), where we find a value of 0.5 ± 0.1, i.e., an interior: surface (i:s) ratio of 1:2.

## 3 Discussion

The structure of native plant cellulose has been studied by a wide range of techniques for decades.^25,37,68–70^ However, despite this, the microfibril structure remains to be fully elucidated, with its core structure, microfibril size, and habit still widely disputed.^9,11,15^ In this work, we have studied a range of samples and have used two-dimensional high magnetic field MAS NMR of never-dried cell walls to resolve some of the misinterpretations and inconsistencies that have arisen partly due to the overlap of signals from the cell wall components using 1D NMR. We have taken several steps to achieve the high resolution observed in the spectra we acquired. We only used never-dried cell walls because it is observed in softwoods^53^ and hardwoods^36^ that dried plant samples give significantly broader NMR spectra. We have also acquired data for sufficiently long in the indirect dimension, alongside utilizing some of the highest magnetic fields available to achieve higher resolution. The use of hCNFs in this work also increased the apparent resolution of the cellulose spectrum, in part by removing components such as lignin and hemicelluloses such as xylan that might alter cellulose chemical shifts.

The breadth of studies regarding cellulose crystallinity across plant and material sciences has given rise to terminology that can be confusing, especially when the same terminology is applied to data acquired by different methods. For example, describing cellulose as crystalline or amorphous has different meanings in NMR studies compared to diffraction experiments. Measuring crystallinity of a cellulose sample by NMR is based on a much shorter range order than that probed by diffraction techniques (see also Table S4).^71^ Historically, the glucose residues observed in the two domains of NMR spectra were assigned as crystalline and amorphous cellulose because the characterized crystalline forms of cellulose I have ^13^C chemical shifts in the spectral domain 1 (C4–89 ppm) region.^44,72,73^ Surface residues that give peaks in spectral domain 2 are sometimes described as paracrystalline or amorphous,^27^ leading to the ratio of the C4^1^ and C4^2^ peaks from the 1D NMR spectrum being commonly called (predominantly by material scientists) the crystallinity index (CI). This index is currently routinely used to describe the crystallinity of cellulosic samples alongside crystallinity measured by X-ray diffraction.^44,72^ The measured values of crystallinity by NMR versus diffraction techniques are always significantly different, although the general trends across a range of samples tend to be consistent.^44^

In our work, we find that the line widths of the signals from the six glucose environments within the surface and core of the microfibril in the hCNF sample are very similar, e.g., the C6 region of the CP refocused INADEQUATE spectrum of the xylanase-treated hCNFs shown in Figure 5. Table S1 summarizes the observed linewidths. NMR chemical shifts are sensitive only to short-range structure (<∼5 Å), and so the similar narrow linewidths indicate that the glucose environments contributing to both domains have similarly high short-range order. While some narrowing could occur due to mobility (dynamic disorder), the amorphous cellulose which has no long-range order would have a substantially broad NMR line width arising from static disorder. Therefore, the microfibril core and surface glucose residues cannot be divided simply into crystalline and amorphous cellulose on an NMR scale. There is, however, a weak (<10%) broad signal in the 1D CP MAS spectrum of poplar stem, seen in Figure 4a underlying the domain 2 region that is removed in the production of the xylanase-treated hCNFs sample. Some of this less ordered component is likely to be lignin as well as xylan, but it could also include a more disordered portion of cellulose that is known to be easily hydrolyzed.^74^ We note that this disordered material in the intact wall is lost during isolation of the hCNFs, calling into question the belief that microfibrils have substantial regions of amorphous cellulose interspersed along each fibril between crystalline domains, i.e., the fringed-fibril model.^16^ Therefore, while there might be a small component of less ordered cellulose giving rise to overlapping signals in the domain 2 region in cell walls and processed lignocellulose, the term amorphous cellulose does not accurately describe the entire origin of this NMR signal when referring to cellulose microfibrils. We further note that, depending on the sample, the ratio of spectral domain 1 to domain 2 signals could be influenced by the microfibril dimension, as well as the presence of any signal from other cell wall components, which does not necessarily arise from cellulose (e.g., lignin and hemicelluloses). Hence, the traditional CI as measured by NMR is a misnomer; it does not measure cellulose crystallinity.

Some groups have designated all spectral domain 1 glucose environments as arising from the interior of the microfibril, with a structure that is a mixture of the two cellulose I allomorphs or even proposing a distinct structure.^15,48,50^ We emphasize that high-resolution MAS NMR spectra as presented in this work have been needed to resolve signals from these domain 1 residues to substantially test these hypotheses. The ^13^C NMR chemical shift is very sensitive to the local environment, allowing us to distinguish differing conformations, such as 2-fold and 3-fold xylan structures^34^ as well as changes in hydrogen bonding. The 2D solid-state NMR spectra of several samples presented here have allowed distinct and recurring glucose residues in the cellulose microfibrils to be resolved and ^13^C NMR chemical shifts to be fully assigned. In contrast to previous work on primary cell walls,^54^ we found that native plant cellulose has just six major glucose environments, with only three of these contributing to the spectral domain 1 region. Within domain 1, we have now unambiguously assigned the glucose environments a and c as cellulose Iβ, since the ^13^C NMR shifts of our tunicate sample, as well as those of cellulose Iβ given by Brouwer and Mikolajewski, are identical to those found here.^57,58^ We confirm, using the 30 ms ^13^C CP MAS PDSD NMR spectra of our samples, that the C1 ^13^C chemical shift of the two glucose residues of tunicate cellulose Iβ had previously been misassigned by Kono, as was recently also suggested by Brouwer and Mikolajewski.^57,58^ With this assignment, the differences in the two residues are now more consistent with DFT calculations of cellulose Iβ origin and center chain environments,^54,66^ allowing us to assign origin chains to site a and center chains to site c (Table S2). Note that, since these core environments have the ^13^C chemical shifts of cellulose Iβ, they must be surrounded by chains packed in a cellulose Iβ fashion, and hence, we deduce that the microfibril is entirely cellulose Iβ.

This assignment of the cellulose Iβ structure of the native plant cellulose microfibril is consistent with the clear lack of cellulose Iα signals in the 2D MAS NMR spectra. The assertion that native plant cellulose is a mixture of the two cellulose I allomorphs originally arose by comparing 1D NMR spectra of native plant cellulose with those of the two biological crystalline allomorphs of cellulose.^30^ It was found that there was an additional component in the domain 1 C4 region, which meant that the origin and center chains were not in a 1:1 ratio as expected of a cellulose Iβ crystal. By adding a small amount of cellulose Iα structure to the simulation of the 1D NMR spectra, the resulting fit appeared good.^30,68^ However, if there were cellulose Iα present in plants, we would expect to observe two equal intensity Iα glucose environments contributing to domain 1 of the 2D spectra. On the contrary, we found that there is only one further major glucose environment, b (see Figure 6b). This glucose environment b has similar C1 and C4 shifts to one of the glucose residues of cellulose Iα, perhaps leading to the earlier misinterpretation of the 1D spectra. We have now conclusively shown that the microfibril is solely cellulose Iβ, and so cellulose modeling, computational studies, and interpretation of FTIR and diffraction patterns should be based solely on this Iβ structure.

Glucose residues with ^13^C chemical shifts in domain 1 (C4 around 89 ppm) have long been considered to reside in the crystalline core of plant cellulose.^43,55^ However we have now shown, using water-edited experiments, that the third major glucose environment in spectral domain 1, site b, is a residue that is in close proximity to water, which could indicate that it is not in the microfibril core with sites a and c and could be on the surface of the microfibril. We previously suggested^34^ that there could be a surface component in the domain 1 region and recently this view has been supported by the work of Addison et al.^36^ Calculations have shown that a change in C6 hydroxymethyl conformation from *tg*, to *gt/gg* changes the C4 ^13^C chemical shift by ∼5 ppm.^18,47^ Adoption of the *tg* conformation is likely to occur where the C6 is facing toward the inside of the microfibril where the orientation is fixed by hydrogen bonding to other glucosyl residues rather than water. Therefore, our experiments indicate that the glucose residue in environment b is an hydrated site, which could be a surface glucan chain with a *tg* conformation, perhaps due to the C6 carbon facing inward toward other chains in the microfibril. The previously proposed models requiring multilayered microfibril environments^75^ or bundling^43^ to generate additional interior chains are not necessary to explain the major glucose environments within spectral domain 1. It is now clearly more appropriate, for samples containing cellulose I, to describe spectral domain 1 glucose residues as possessing *tg* C6 hydroxymethyl conformation and that spectral domain 1 reflects neither only the crystalline cellulose nor the exclusively interior of the microfibril. The differing assignments of spectral domain 1 and spectral domain 2 has led to a range of different models to illustrate the widely debated structure of native plant cellulose.^9,37,43,54,55^ While some models are able to explain some of the phenomena observed in cellulose, there is no overarching model that is able to describe cellulose in a wide range of materials. Previous studies have used the ratio of C4^1^ and C4^2^ from 1D NMR spectra as a measure of microfibril size, which has resulted in variable estimations such as 18–24 chains in a microfibril or alternatively microfibril bundling models.^9,43^ The bundling models of the cellulose microfibril were suggested because the amount of domain 1 was used as a measure of the fibril interior, giving a high proportion that is inconsistent with 18–24 chain models, unless they aggregate to reduce the surface. As we now know, domain 1 does not arise solely from core Iβ sites, thus the ratio of C4^1^ and C4^2^ will give an overestimate of the interior of the microfibril and thus overestimate the microfibril size. If there is a single fibril type in the sample, the ratio of the two domains could still be utilized for characterization as it is loosely related to the volume of the microfibril, but care is needed in using this interpretation. In this work, we have provided a more accurate measure of the interior to surface ratio (i:s) for the microfibril of poplar wood, which was found to be ∼0.5 ± 0.1, i.e., i:s = 1:2. We also gain insight into the proportions of the environments in the microfibril; namely, origin (a) and center (c) chains are similar in proportion (1:1 ratio). Reliable quantification of site b was not possible in this work, as site b is only resolvable in certain regions of the 2D NMR spectra. The signal intensity seen in two-dimensional NMR spectra can be dependent on other factors, such as mobility as well as quantity. Nevertheless, the quantification of the interior to surface ratio is only reliable due to the C1 region intensities not varying during the 1D DQ-filtered experiment, as seen in Figure S11. This is not necessarily true for other regions of the DQ filter spectrum or regions of the CP PDSD spectrum. However, across all 2D NMR spectra of the hCNFs of poplar wood, the relative amount of site b compared to that of site a or c is consistently less. Quantification via deconvolution of 1D spectra has not been possible for this work, as the spectral overlap means small changes in linewidths during fitting can cause large changes in the relative amounts of different environments. Given the constraints on the microfibril dimensions from biophysical measurements, the upper limit is a microfibril with around 18–24 chains; however, there are several habits that could fulfill these criteria.

It should also be noted that NMR is a bulk technique and the spectra and quantification of core to surface are the result of an average of many microfibrils, so care is needed in the interpretation. However, one could assume that the most common microfibrils present in the material will dominate the spectra. The ratio of i:s = 1:2 is consistent with an 18-chain microfibril with a habit that has 6 interior chains and 12 surface chains, but it could also be consistent with a 24-chain microfibril with 8 interior and 16 surface chains. Thus, for the xylanase-treated hCNFs of poplar wood, this would mean that for a slice through an 18-chain average microfibril, there are three of each site a and c chains and hence potentially 2 chains of site b (since b < a and c, see Figure 6) with the remaining 10 chains being domain 2 environments (f, g, and j). The surface and interior ratios alone are not sufficient to give a value for the size of the microfibril. As shown in Figure 10, there are several different-sized microfibrils that could have a ratio close to 1:2 within the error. Hence, further understanding of the relative proportions of the different glucose environments reduces the possible cellulose microfibril habits. For example, two potential 18-chain habits 234432 and 34443 have the observed interior:surface ratio of 0.5. However, given that the cellulose Iβ structure has origin (a) and center (c) chains in alternating sheets, the 34443 with three core layers may not have equal amounts of sites a and c. However, the habit 234432 would fit this constraint.

**Figure 10:**
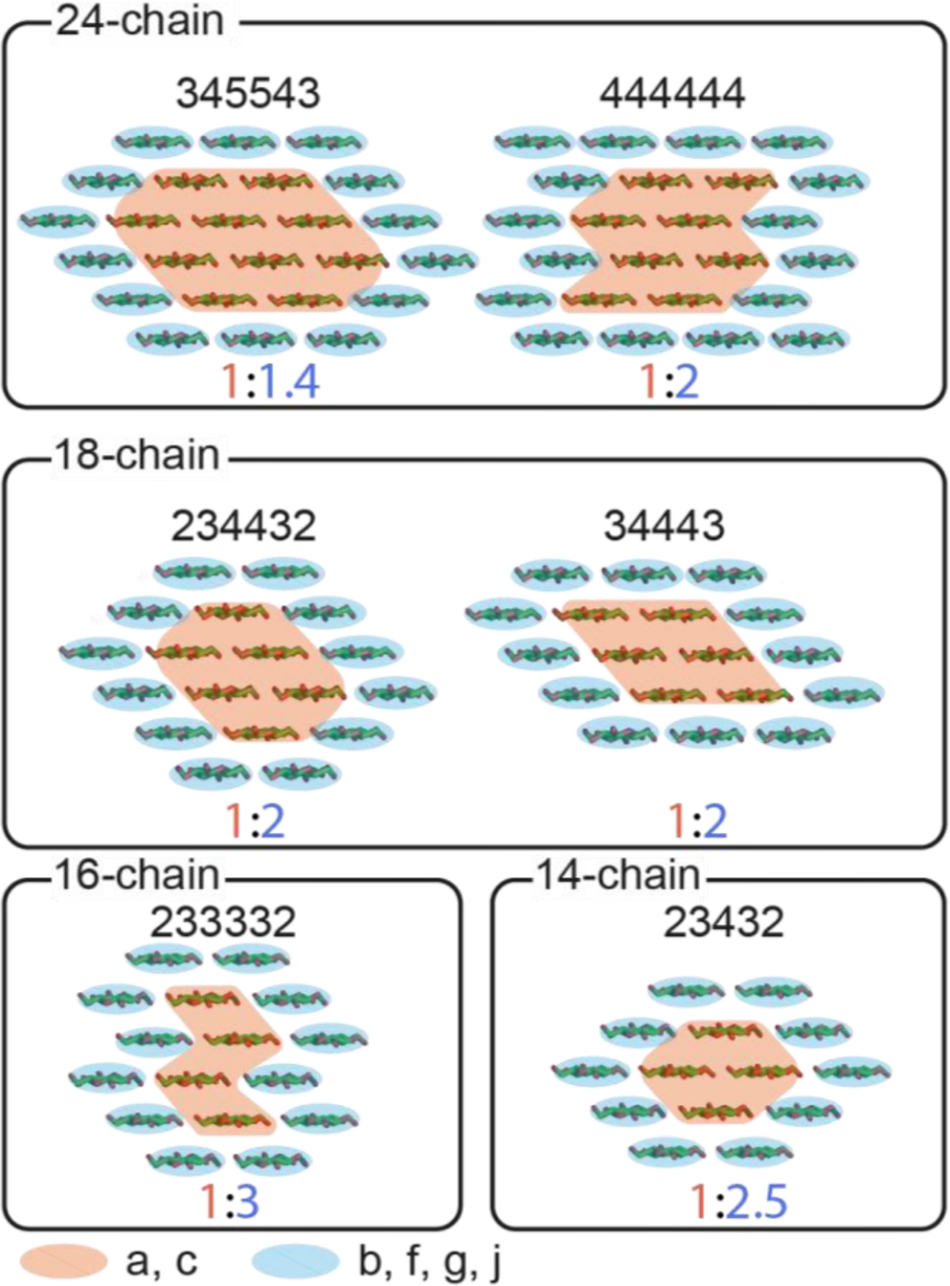
Possible cellulose fibril habits that fit close to the interior to surface ratio of 1:2, within the error as determined here by ^13^C MAS NMR, for the xylanase-treated hCNFs of the poplar wood.

While we cannot yet propose a definitive microfibril structure based on the NMR data, when we understand where site b and the spectral domain 2 environments reside, i.e., in the hydrophilic and hydrophobic surfaces of the cellulose microfibril, their relative proportions will provide a fingerprint of the average cellulose microfibril structure within that plant sample. The relative amount of site b compared to core chain sites a and c for isolated microfibrils is likely to vary with any changes in the microfibril type, size, habit, and hemicellulose interactions in different plants. Therefore, the relative proportions of the six major glucose residue environments will be an important diagnostic tool for discovering cellulose variability, studying cellulose interactions, and detecting changes during degradation or industrial processing of wood and other biomass. For example, in a study of cotton by Kirui,^37^ it was found that there is relatively more of the core glucose environments a and c compared to the surface environments (b, f, g, and j), which is consistent with cotton having a significantly larger fibril structure.^76^ However, we find that the relative amounts of site a and c are less than site b in the *Brachypodium* sample we have studied in this work (Figure 3), suggesting that there is either a smaller microfibril on average or a different habit with fewer core chains compared to those proposed for poplar wood. These differences indicate distinct variability in the cellulose structure across different plant cell walls. The use of hCNFs from different plant cell walls could help distinguish whether these differences arise due to cell wall interactions or changes in the size or habit of the average cellulose microfibril.

## 4 Conclusion

We have clarified that plant cellulose microfibrils have a cellulose Iβ structure: we do not detect any cellulose Iα. Moreover, we find no evidence for amorphous cellulose in the microfibrils, with the surface glucose residues being well ordered. We show that a spectral domain 1 environment (site b), previously thought to be interior, is near water and could instead be a surface glucose residue. This work changes widely held interpretations of solid-state ^13^C MAS NMR spectra, since previous interpretations of sample crystallinity and surface to core ratios are shown to be flawed. The proportion of spectral domain 1 to domain 2 is better understood to reflect the ratio of C6 hydroxymethyl group conformations *tg* vs *gt/gg* and so is influenced largely by whether the residue is hydrogen bonded to another cellulose chain or to water. Our results now also provide a strong basis for understanding cellulose microfibril surfaces, as we have shown that there could be four major types of surface residues, which likely reflect, in a manner to be determined, the different hydrophobic and hydrophilic surfaces of microfibrils. The exciting prospect now arises of a more complete description and understanding of cellulose microfibril surfaces, their interactions, and variations in both biomaterials and plant cell walls.

## 5 Methods

### 5.1 Sample Production and Preparation

The poplar stem *Populus tremula* × *tremuloides* used in this work was grown from poplar shoot cuttings, which were allowed to root for 2 weeks before being transferred into the growth chamber. Here, the saplings were grown in a ^13^CO_2_ atmosphere^34,77^ for ∼3 months to provide ∼97% ^13^C-labeled material that is predominantly secondary cell wall. Only wood from the lower (woody) never-dried stems was used after the removal of bark and cambium. The growth of spruce and *Brachypodium* was as described in Terrett et al. and Tryfona et al., respectively.^52,59^

To prepare hCNFs,^56^ the never-dried wood after removing bark and cambium was first delignified four times with 3% peracetic acid (PAA; 0.35 g pure PAA/g dry material, pH 4.8) at 85 °C for 45 min without stirring. Between cycles, used PAA was decanted, one water wash was done, and 3% PAA was added. The delignified material (holocellulose) was washed with water after the fourth cycle until the conductivity was below 10 S/cm. The holocellulose was blended for 2 min to form a homogeneous slurry. The holocellulose dispersion (0.1 wt %) was blended for 30 min in a Vitamix A3500i blender (Vitamix, US) to make a polydispersion. hCNFs were recovered as the supernatant from the polydispersion by centrifugation at 5000 rpm for 15 min. hCNFs were concentrated by filtration onto a PVDF membrane, and the wet cake was then freeze-dried and rewetted into the NMR rotor.

To remove the xylan, the hCNF suspension was digested with GH10, CE4, and GH115 in 0.1 M ammonium acetate buffer, pH 6.0, at 30 °C on a shaker (120 rpm) at a solid loading of 0.075 wt %. The enzymes were denatured by heating the reaction mixture to 100 °C for 10 min after 20 h. Subsequently, the mixture was centrifuged at 10,000 rpm for 20 min at 4 °C. A PVDF membrane with a pore size of 0.1 μm was employed to filter the clear supernatant. The retentate from the filter membrane (if collected) and the bottom fraction from centrifugation were combined and redispersed in an ethanol–water mixture at a concentration of 65% (v/v). The ethanol/water mixture underwent centrifugation and filtration once, while the water washing was repeated three times. Lastly, the water-washed undigested fraction was resuspended in water at a concentration of 0.1–0.12 wt % and blended in a Vitamix A3500i for 2 min. The suspension that resulted was referred to as xylanase-treated hCNFs. The xylanase hCNF dispersion was freeze-dried and rewetted before being packed into the NMR rotor.

The never-dried ^13^C-enriched poplar wood was debarked and cut into small pieces of ∼1–2 mm size with a razor blade, then packed into a 3.2 mm MAS zirconia NMR rotor while removing excess water.

Tunicate (*Halocynthia roretzi*) was purchased from a local supermarket (Yoshiike, Japan). The cellulose was purified according to the method described previously.^78,79^ After the entrails were removed, the tunicate mantle was deproteinized and bleached by four cycles of alkaline treatment with 5% KOH (Fujifilm Wako, Japan) at 37 °C and oxidation with sodium chlorite (Fujifilm Wako, Japan) at 37 °C and pH 5, respectively. The bleached tunicate mantle was homogenized with a Vitamix V1200i blender (Vitamix, US) for 2 min at the top speed and was subsequently acid hydrolyzed with 50% sulfuric acid at 40 °C for 8 h. The sediment was collected by filtration and thoroughly washed with deionized distilled water, followed by homogenization to gain an aqueous suspension of cellulose microcrystals. For ^13^C NMR, tunicate cellulose was centrifuged at 20,000*g* for 30 min to provide a more concentrated cellulose sample.

### 5.2 TEM

A Thermo Fisher Scientific (FEI) Talos F200X G2 microscope operating in scanning mode at 200 kV was used to obtain the TEM images.

### 5.3 Solid-State NMR

Solid-state NMR experiments of poplar wood and the xylanase-treated hCNFs were acquired on a Bruker 1 GHz AVANCE NEO solid-state NMR spectrometer operating at ^1^H and ^13^C Larmor frequencies of 1000.4 and 251.6 MHz, respectively, using a 3.2 mm E^Free^ triple resonance MAS probe. All experiments were conducted at an input gas temperature of 10 °C and an MAS frequency of 12.5 kHz with a recycle delay of 2 s. The ^13^C chemical shifts were determined using the carbonyl peak at 177.87 ppm of l-alanine as an external reference, with respect to tetramethylsilane. This referencing was confirmed to correspond with referencing to the adamantane CH_2_ peak at 38.48 ppm^80^ to ensure direct comparison with Brouwer and Mikolajewski.^23,58^ The 90° pulse lengths were typically 3.2 (^1^H) and 4.0 μs (^13^C). Cross-polarization from ^1^H to ^13^C was achieved using ramped (70–100%) ^1^H radiofrequency amplitude^81^ and a contact time of 1 ms. SPINAL-64 decoupling was applied at an ^1^H nutation frequency of 70–80 kHz during acquisition and during the indirect dimension of 2D experiments.^82^ Sign discrimination in the indirect dimensions of the 2D experiments was achieved using the States-TPPI method. For the principal assignments of cellulose environments, a CP refocused INADEQUATE ^13^C double-quantum (DQ)–^13^C single-quantum (SQ) correlation experiment was used, whereby the evolution of DQ coherence for directly bonded carbons within the same glucan ring is correlated with directly observed SQ coherence.^63,83^ The acquisition time in the indirect dimension was 6.67 ms, with a spectral width of 37.5 kHz and 128 acquisitions per *t*_1_ FID. The echo (τ–π–τ) duration, τ, was 2.24 ms, giving a total echo time of 8.96 ms. Both intra- and intermolecular contacts were probed using 2D ^13^C–^13^C CP MAS proton-driven spin diffusion (PDSD) experiments with mixing times of 30 to 400 ms.^84^ The acquisition time in the indirect dimension (*t*_1_) of the CP MAS PDSD experiments was 5.5–8.1 ms. The spectral width in the indirect dimension was 37.5 kHz with at least 64 acquisitions per *t*_1_ FID. The proximity of water to different cellulose environments was probed using both a water-edited ^13^C–^13^C 30 ms CP MAS PDSD experiment and a water-edited CP MAS refocused INADEQUATE experiment. These water-edited experiments are based on the normal CP MAS refocused INADEQUATE and CP MAS PDSD experiment; however, before the CP contact time, there is an ^1^H *T*_2_ filter (spin echo) to remove signal arising from directly attached protons, followed by a delay to allow for spin diffusion from the water protons to its near neighbors.^85^ The CP parameters were as stated above. The acquisition time for the water-edited ^13^C–^13^C 30 ms CP MAS PDSD experiment in the indirect dimension was 4.8 ms with a spectral width of 37.5 kHz and 576 acquisitions per *t*_1_ FID. The total ^1^H *T*_2_ filter was 320 μs, followed by a spin diffusion delay of 2 ms. For the water-edited CP refocused INADEQUATE, the acquisition time in the indirect dimension was 4.7 ms, with a spectral width of 28.5 kHz and 960 acquisitions per *t*_1_ FID. The total ^1^H *T*_2_ filter was 280 μs, followed by a diffusion delay of 2 ms. All 2D spectra were processed with Fourier transformation into 8 K (*F*_2_) × 2 K (*F*_1_) points with exponential line broadening of 20–50 Hz in *F*_2_ and cubed sine bell processing in *F*_1_ using Bruker Topspin v.3.6. Contour levels are 1.1 × 1.1 throughout. The minimum contour is chosen to show the desired features. Key experimental details are summarized in Table S3.

## Supplementary Material



## Data Availability

Raw NMR data files will be available from http://wrap.warwick.ac.uk/id/eprint/195004/

## Notes

Additional comparisons of NMR spectra of poplar wood and xylanase-treated hCNFs of poplar wood to support the main NMR figures, TEM images of the xylanase-treated hCNFs of poplar wood, a table of ^13^C chemical shifts of sites a, b and c compared to cellulose Iα and Iβ NMR shifts, a table of detailed experimental parameters for all the NMR experiments, a table of definitions, and a table of acronyms (PDF)

## ∥

Faculty of Biology-Oriented Science and Technology, Department of Science and Technology on Food Safety, Kindai University, 930 Nishimitani, Kinokawa, Wakayama 649-6493, Japan

## ⊥

Department of Chemistry and Biochemistry, The Ohio State University, College of Arts and Sciences, 151 W. Woodruff Ave., Columbus, OH 43210, U.S.A.

## #

Deceased on 17 June 2025
