# Supplementary material for: Using Solid-State NMR to Understand the Structure of Plant Cellulose
Source: J Am Chem Soc. 2025 Dec 11;147(51):47223–36. doi: 10.1021/jacs.5c14452 (PMC12752684; doi:10.1021/jacs.5c14452)
Supplement: Supplementary file 1 [file ja5c14452_si_001.pdf]

Supplementary Information- Using solid-state NMR to understand the structure of plant cellulose

Rosalie Cresswell[1], Parveen Kumar Deralia[2], Yoshihisa Yoshimi[2 *], Tomohiro Kuga[2,3], Alberto Echevarría-Poza[2], W. Trent Franks[1 #], Steven P. Brown[1], Ray Dupree[1], Paul Dupree[2]

1. Department of Physics, University of Warwick, Coventry CV4 7AL, UK;

2. Department of Biochemistry, University of Cambridge, Hopkins Building, The Downing Site, Tennis Court Road, Cambridge CB2 1QW, UK

3. Department of Biomaterial Sciences, Graduate School of Agricultural and Life Sciences, The University of Tokyo, 1-1-1 Yayoi, Bunkyo-ku, Tokyo 113-8657, Japan.

* Now at Faculty of Biology-Oriented Science and Technology, Department of Science and Technology on Food Safety, Kindai University, 930 Nishimitani, Kinokawa, Wakayama 649-6493, Japan.

# Now at Department of Chemistry and Biochemistry, The Ohio State University, College of Arts and Sciences, 151 W. Woodruff Ave., Columbus, OH 43210, U.S.A.

Corresponding author: Paul Dupree - pd101@cam.ac.uk

Contents

Supplementary Figures

SI Figure 1: 30 ms ^{13}C 2D CP PDSD spectrum of poplar wood.

SI Figure 2: TEM images of hCNFs of poplar wood.

SI Figure 3: Comparison of the 1D CP MAS NMR spectra of poplar wood, hCNFs of poplar wood and xylanase-treated hCNFs of poplar wood.

SI Figure 4: Cellulose of poplar wood maintains its structure in the fibrillation process to form hCNFs.

SI Figure 5: Cellulose of poplar wood maintains its structure in the fibrillation process to form hCNFs.

SI Figure 6: The neutral carbohydrate region of the CP refocussed INADEQUATE showing the environments a and c from the xylanase treated hCNFs of poplar wood compared to the NMR shift positions of the two Iβ cellulose glucose units.

SI Figure 7: The C3-C1 and C3-C4 regions of a 30 ms ^{13}C 2D CP PDSD spectrum showing the environments a and c from the xylanase treated hCNFs of poplar wood compared to the NMR shift positions of the two Iβ cellulose glucose units.

SI Figure 8: Neutral carbohydrate region of standard (blue) and water-edited CPMAS PDSD spectra (brown).

SI Figure 9: Comparison of 1D water-edited CP spectra of xylanase treated hCNFs of poplar wood with different ^{1}H T_2 filter times

SI Figure 10: Similar water proximity of the three domain 2 surface glucose environments of cellulose.

SI Figure 11: Determining the interior to surface ratio from the relative amounts of glucose environment b and D2 versus core environments a and c.

Supplementary Tables

SI Table 1: The linewidths of glucose environments in cellulose from the C6 region of the CP INADEQUATE spectrum.

SI Table 2: Comparison of Iα and Iβ cellulose ^{13}C chemical shifts with assigned glucose environments a, b and c from spectral domain 1.

SI Table 3: Summary of solid-state NMR experimental parameters.

SI Table 4: Definitions of key terms used.

SI Table 5: List of acronyms

Supplementary Figures

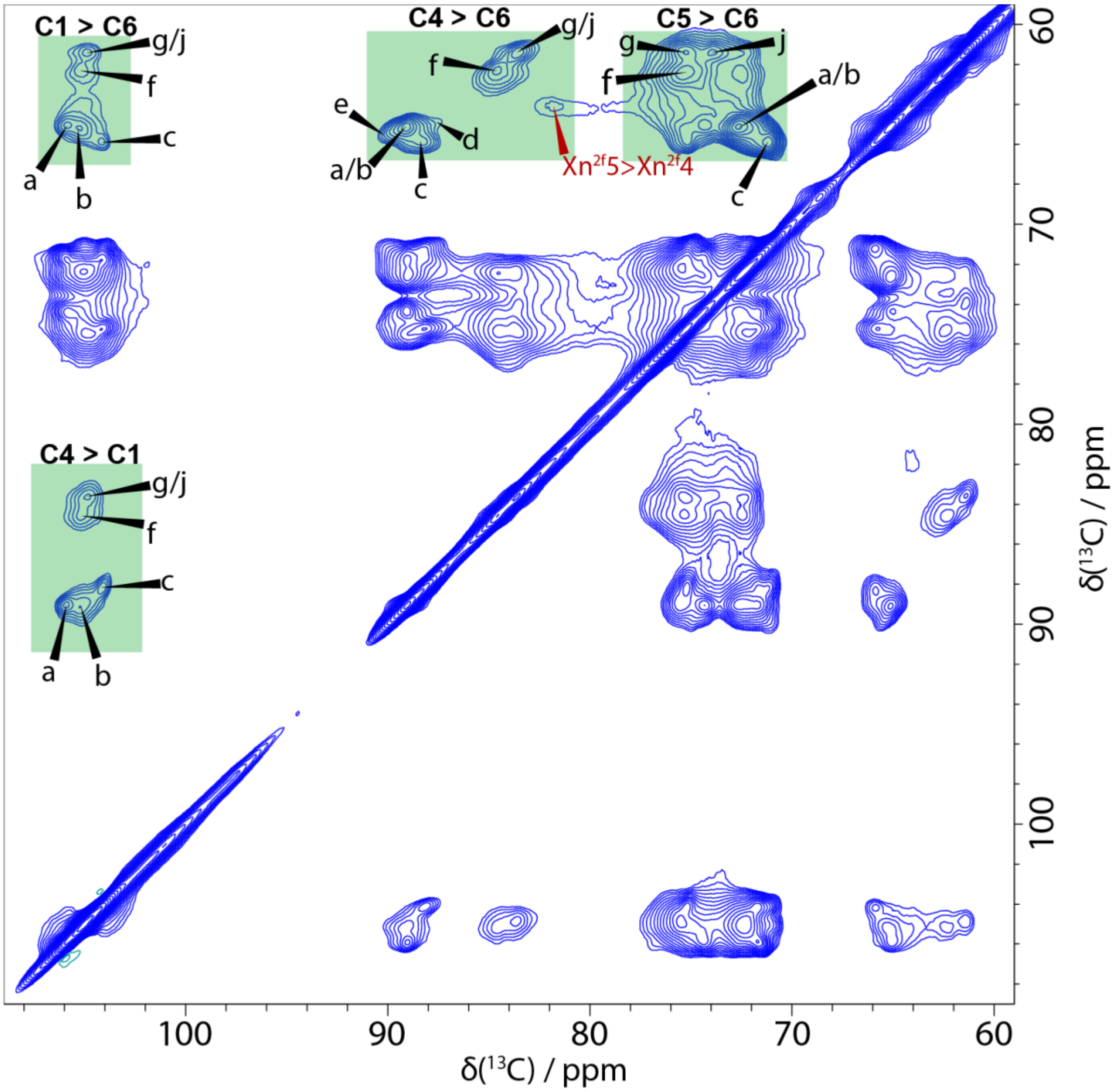

SI Figure 1: 30 ms ^{13}C 2D CP PDSD spectrum of poplar wood. *The main regions of the 30 ms ^{13}C 2D CP PDSD spectrum where different glucose environments in cellulose can be resolved have been highlighted in green. The spectrum was recorded at a ^{13}C Larmor frequency of 251.4 MHz and a MAS frequency of 12.5 kHz.*

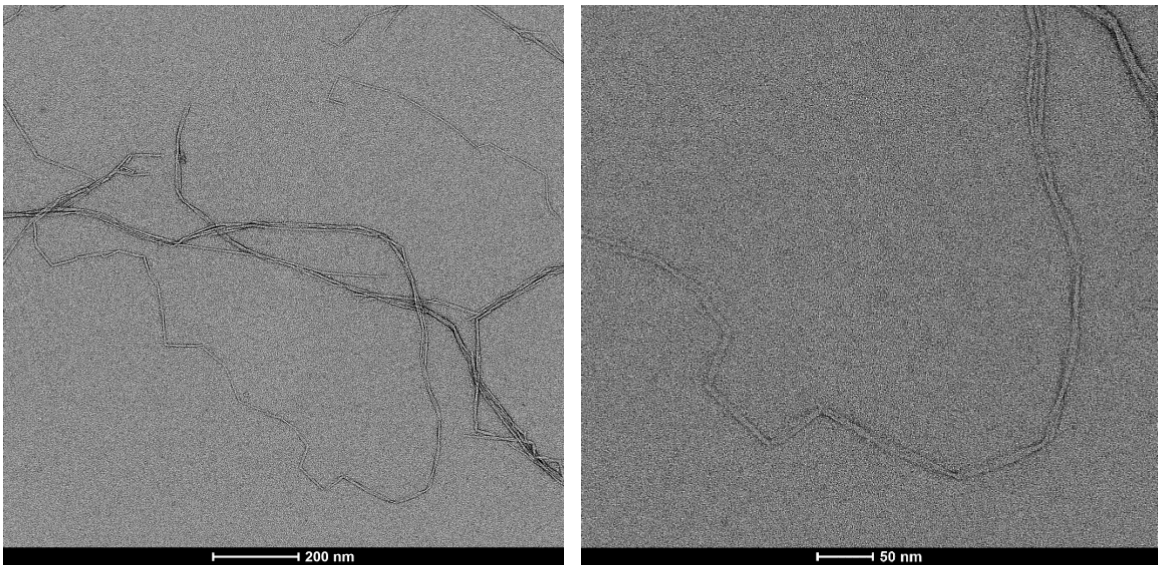

SI Figure 2: TEM images of hCNFs of poplar wood. *The average width of microfibrils is ~3 nm.*

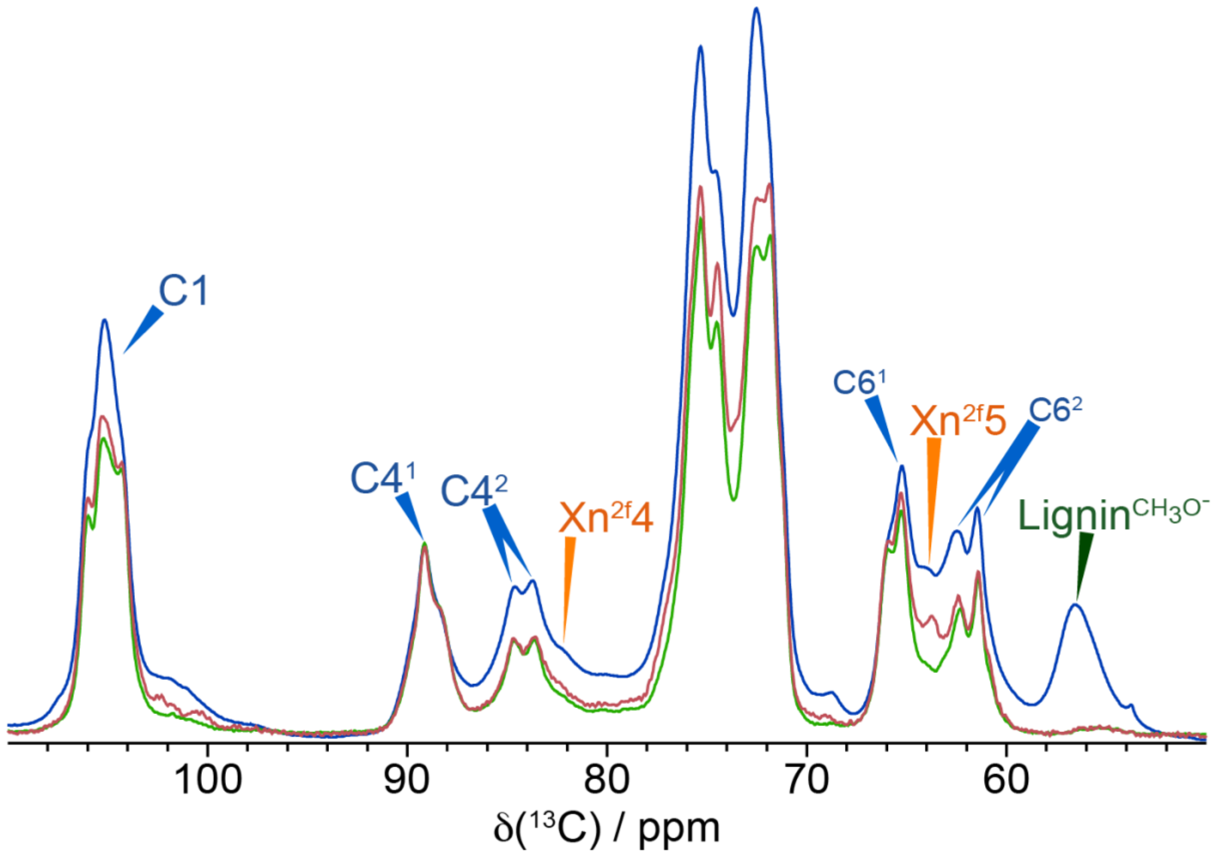

SI Figure 3: Comparison of the 1D ^{13}C CP MAS NMR spectra of poplar wood, hCNFs of poplar wood and xylanase-treated hCNFs of poplar wood. *The comparison (normalised to C4 at 89 ppm) shows the difference in the 1D CP spectrum of poplar wood (blue), hCNFs of poplar wood when lignin and some hemicellulose has been removed (red) and xylanase-treated hCNFs of poplar wood where the majority of xylan has been removed (green). The production of hCNFs results in the removal of the broad background signal whilst the xylanase treatment removes more of the xylan. Spectra were recorded as in SI Figure 1.*

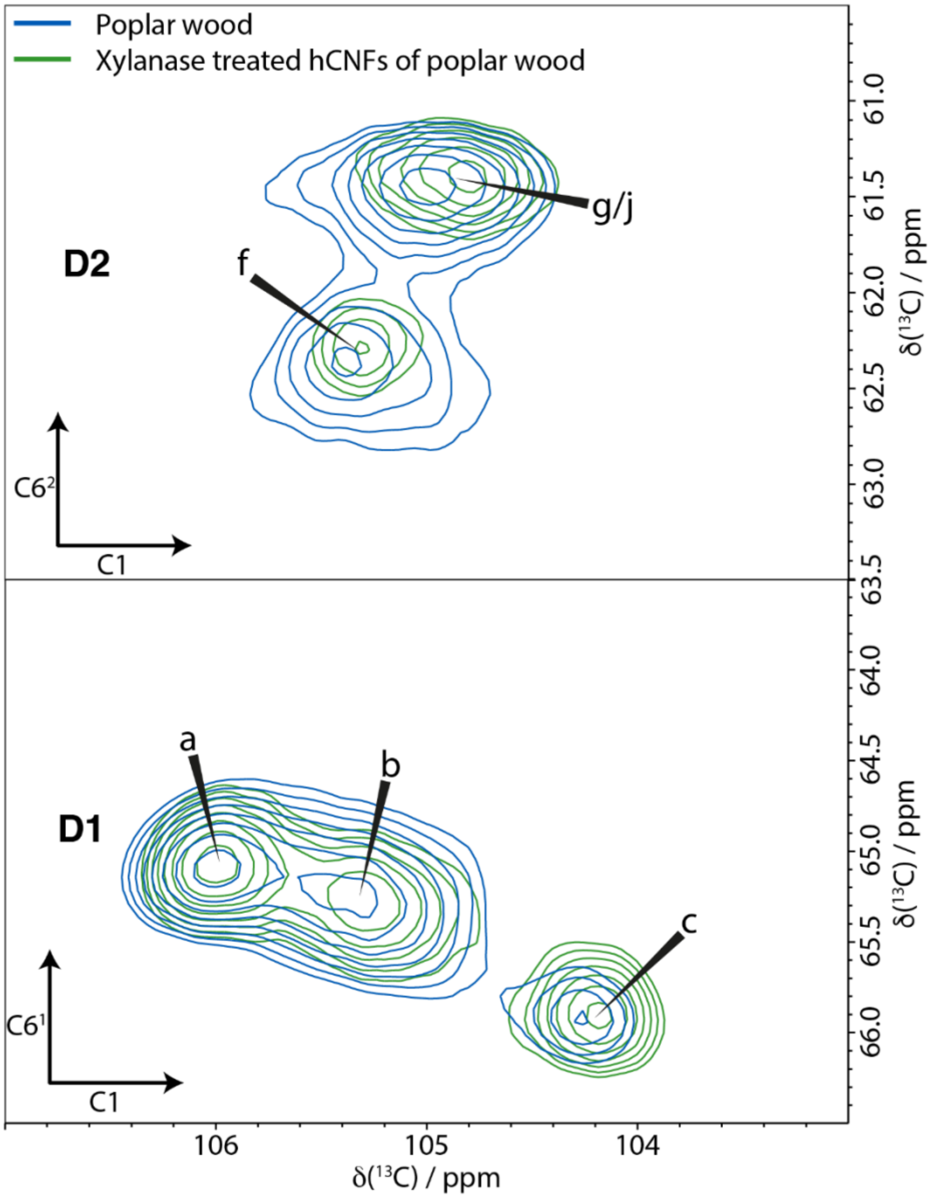

SI Figure 4: Cellulose of poplar wood maintains its structure in the fibrillation process to form hCNFs. *Comparison of the C6-C1 region of 30 ms ^{13}C 2D CP PDSD spectrum of poplar wood (blue) and xylanase-treated hCNFs of poplar wood (green) normalised to the $C6^1$>C1 cross peak of site a. Spectra were recorded as in SI Figure 1.*

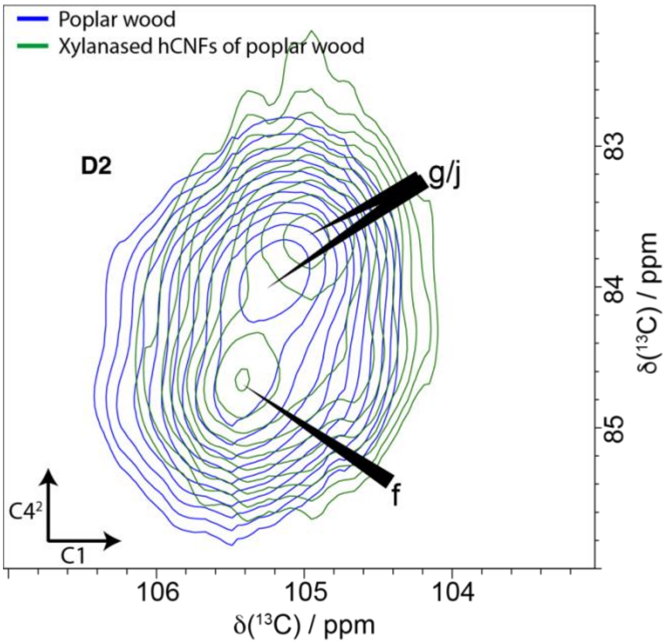

SI Figure 5: Cellulose of poplar wood maintains its structure in the fibrillation process to form hCNFs. *Comparison of the C4^2-C1 region of 30 ms ^{13}C 2D CP PDSD spectrum of poplar wood (blue) and xylanase-treated hCNFs of poplar wood (green) normalised to the C6^1>C1 cross peak of site a. Spectra were recorded as in SI Figure 1.*

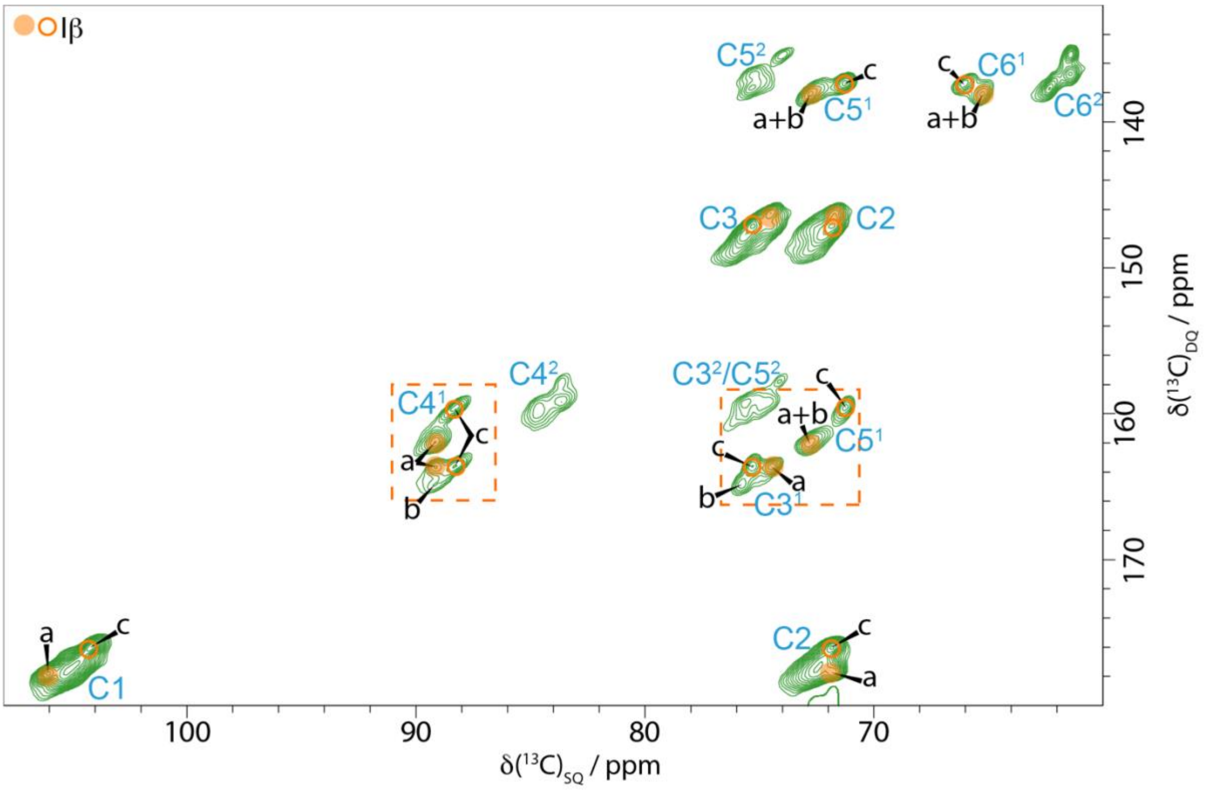

SI Figure 6: The neutral carbohydrate region of the CP ^{13}C refocussed INADEQUATE MAS NMR spectrum (for which Figure 6 in the main text shows the domain 1 C4-C3/C5 region) showing the environments a and c from the xylanase treated hCNFs of poplar wood compared to the NMR shift positions of the two Iβ cellulose glucose units. *Circles are the Kono et al. assignments[21,22] except for C1 which has been exchanged according to our assignments from the CP PDSD MAS NMR spectrum (see SI Fig. 7) with the chemical shifts corrected by Brouwer and Mikolajewski.[23,57,58] This shows that cellulose sites a and c closely match all the corrected assignments for cellulose Iβ. The dashed boxes indicate the regions shown in figure 5. Spectra were recorded as in SI Figure 1.*

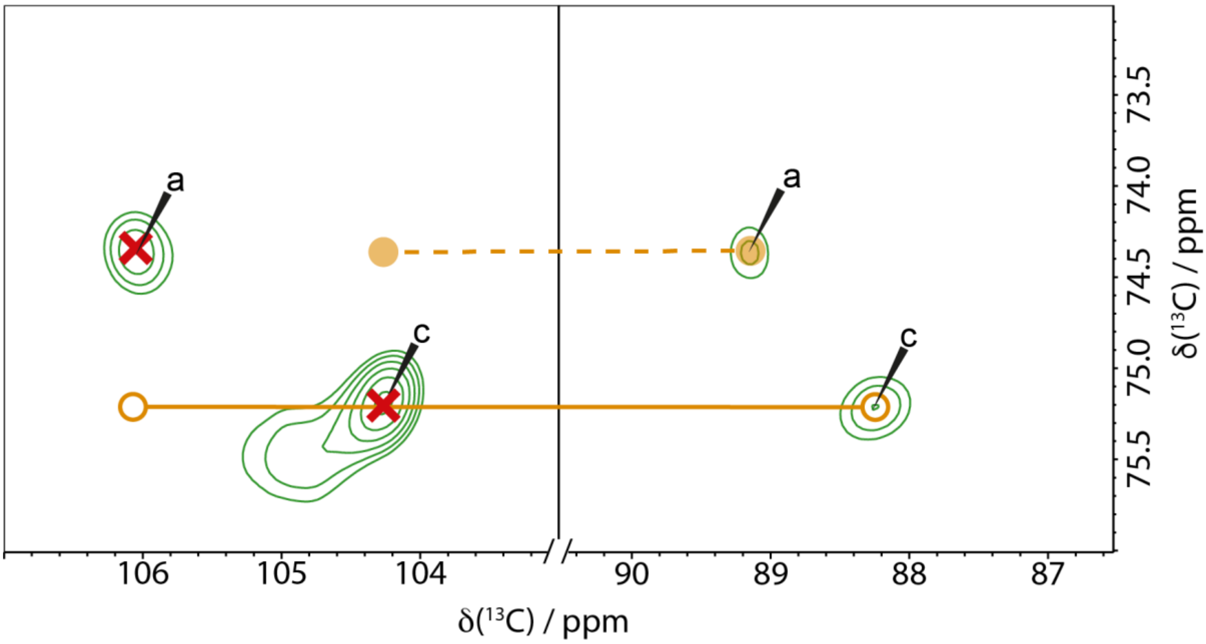

SI Figure 7: The C3-C1 and C3-C4 regions of a 30 ms ^{13}C 2D CP PDSD MAS NMR spectrum showing the environments a and c from the xylanase treated hCNFs of poplar wood compared to the NMR shift positions of the two Iβ cellulose glucose units. *Circles are the Kono et al. assignments[21,22] with chemical shifts corrected by Brouwer and Mikolajewski.[23,57,58] The crosses indicate where the peaks would be if the C1 assignment were switched, as determined in this work, showing a and c closely match the corrected assignments for cellulose Iβ. Spectra were recorded as in SI Figure 1.*

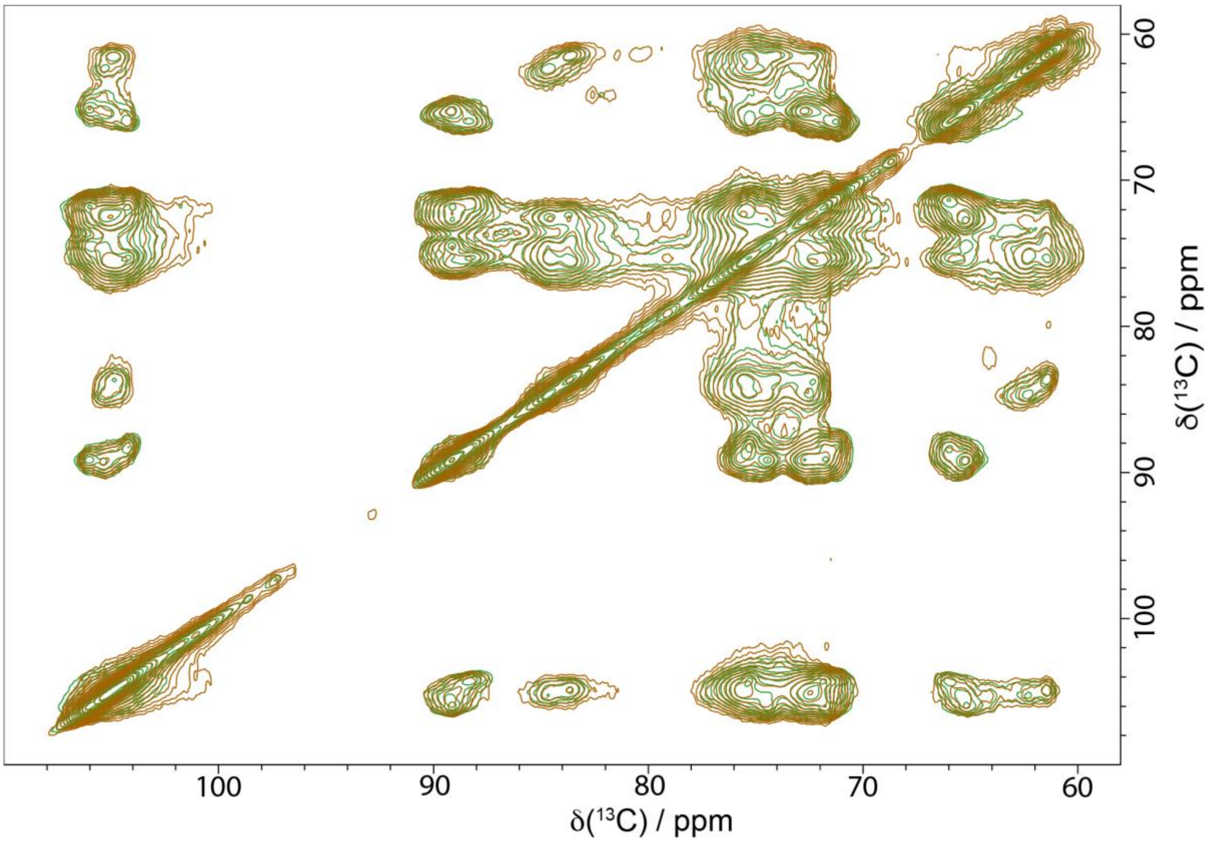

SI Figure 8: Neutral carbohydrate region of standard (green) and water-edited ^{13}C CPMAS PDSD NMR spectra (brown) of xylanase treated hCNFs of poplar wood. *These spectra are normalised to the C1 diagonal peak at ~105 ppm. There is signal for all the cross peaks in both spectra however it is the relative intensities of these cross peaks that is most informative and is highlighted in Fig. 6.*

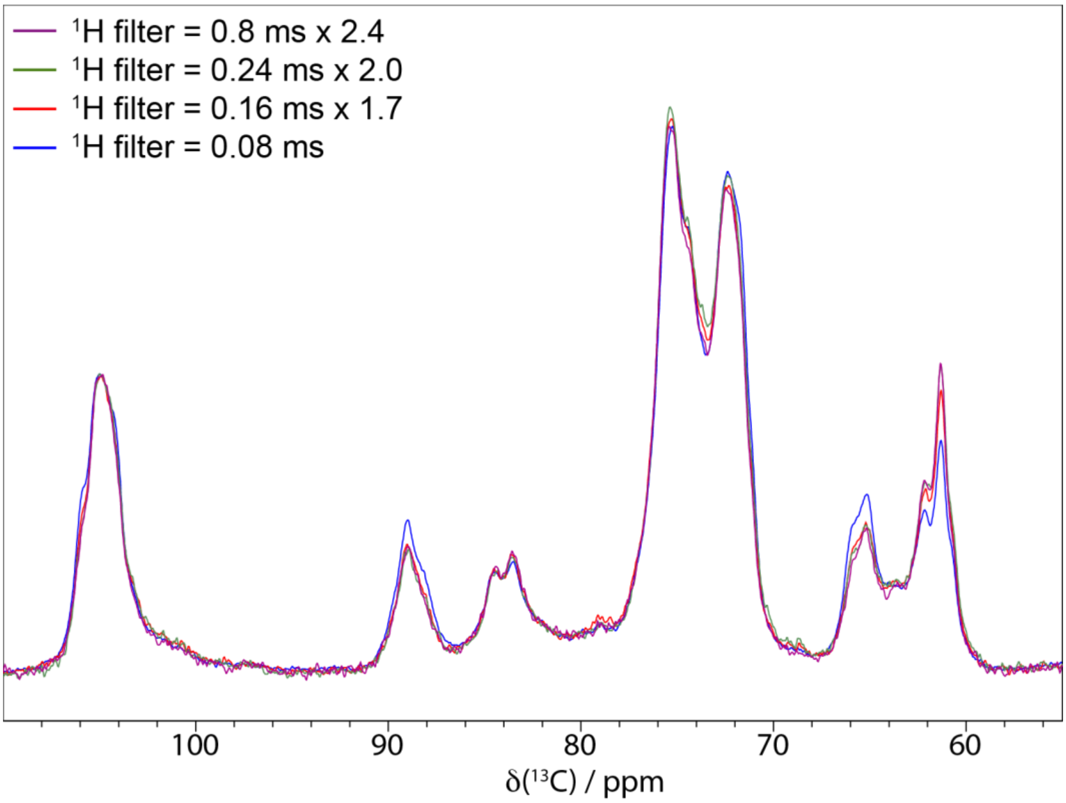

SI Figure 9: Comparison of 1D water-edited ^{13}C CP MAS NMR spectra of xylanase treated hCNFs of poplar wood with different ^{1}H T_2 spin-echo filter times (0.08 ms – blue, 0.16 ms – red, 0.24 ms – green, 0.8 ms - mauve) and a spin diffusion time of 2 ms. *The spectra with ^{1}H filter times of 0.8 ms and 0.24 ms overlay almost exactly throughout the spectrum.*

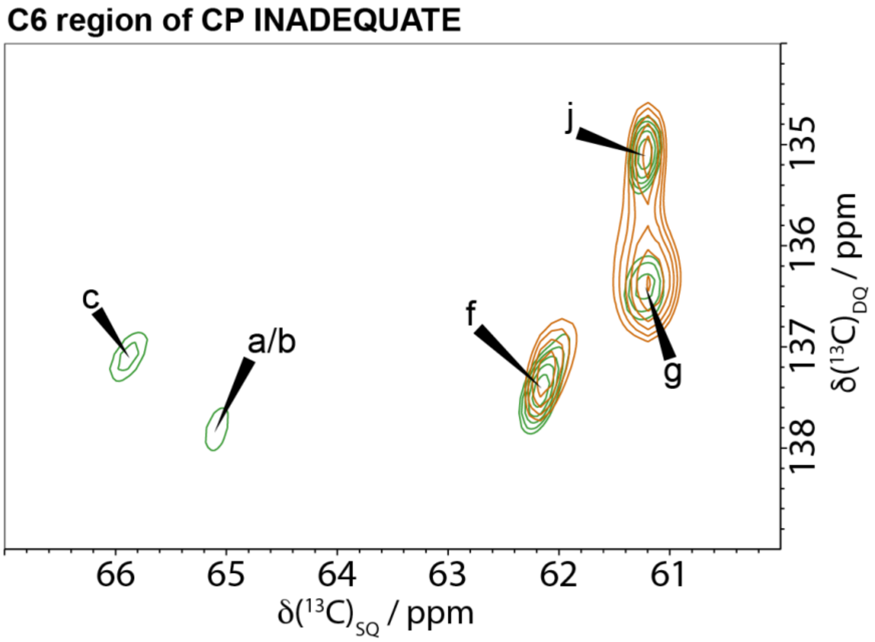

SI Figure 10: Similar water proximity of the three domain 2 surface glucose environments of cellulose. *A comparison of the C6 region of the standard (green) and the water-edited (brown) ^{13}C CP refocussed INADEQUATE MAS NMR spectra of the xylanase treated hCNFs of poplar wood and normalised to site a in the C4[1] region of the CP refocussed INADEQUATE spectrum. Spectra were recorded as in SI Figure 1.*

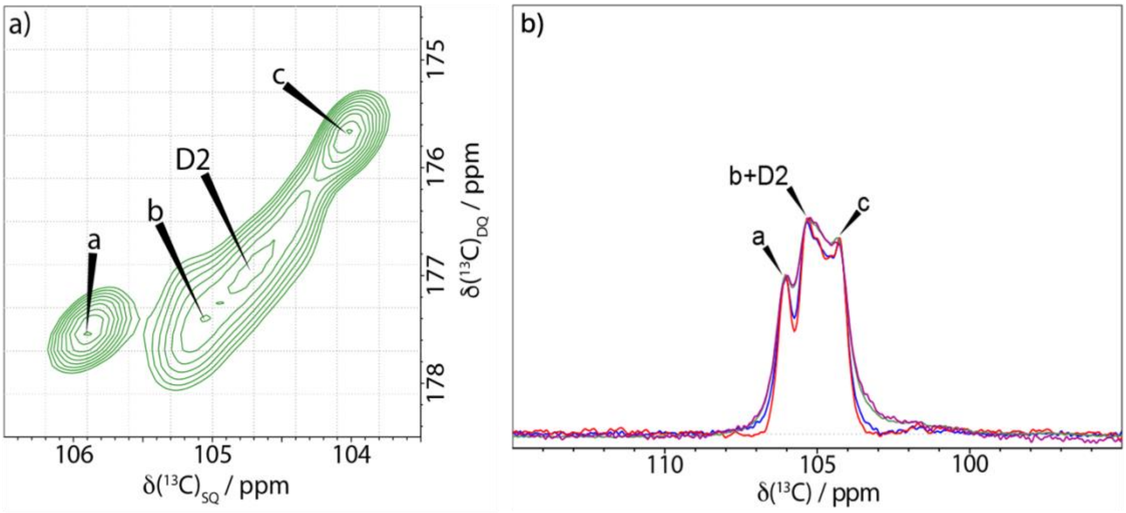

SI Figure 11: Determining the interior to surface ratio from the relative amounts of glucose environment b and D2 versus core environments a and c. *a) C1 region of the ^{13}C 2D CP refocussed INADEQUATE MAS NMR spectrum of the xylanase treated hCNFs of poplar wood. This resolves fibril core sites a and c, allowing integration of the core environments separately from surface environments (b and D2). b) Comparison of the C1 region of the 1D CP (green), 1D quantitative DP (mauve) and two 1D CP Double Quantum filtered (i.e., setting t_1 = 0 in the refocussed INADEQUATE pulse sequence) spectra with an echo time of 0.84 ms (red) and 2.24 ms (blue), of the xylanase treated hCNF sample. Spectra were recorded as in SI Figure 1.*

Supplementary Tables

SI Table 1: The linewidths of glucose environments in cellulose from the C6 region of the ^{13}C CP refocused INADEQUATE MAS NMR spectrum of xylanase-treated hCNFs of poplar stem (see Figure 5 of the main text).

Glucose environment	Linewidth C6 region CP INADEQUATE
a+b†	0.57 ppm
c	0.49 ppm
f	0.57 ppm
g	0.46 ppm
j	0.41 ppm

† The peak labelled a+b has a larger linewidth as it has two components that are ~0.2 ppm different in their NMR shift. Site f has a similar width to the peak for sites a + b and is elongated suggesting it could also be two environments which have yet to be resolved.

SI Table 2: Comparison of Iα and Iβ cellulose ^{13}C chemical shifts with assigned glucose environments a, b and c from spectral domain 1.

	C1	C2	C3	C4	C5	C6
**Cellulose Iα**						
Glucose Unit 1 (Down) †	105.6	72.2	74.6	89.4	73.1	65.7
Glucose Unit 2 (Up) †	105.5	71.2	75.1	90.3	71.3	65.8
**Cellulose Iβ**						
Glucose Unit 1 (Centre) †	106.1	71.7	75.3	88.4	71.4	66.0
Glucose Unit 2 (Origin) †	104.4	71.7	74.4	89.2	72.9	65.2
**Cellulose Iβ (Corrected)** $						
Glucose Unit 1 (Centre)	104.4	71.7	75.3	88.4	71.4	66.0
Glucose Unit 2 (Origin)	106.1	71.7	74.4	89.2	72.9	65.2
**Native plant cellulose ***						
a* (Cellulose Iβ origin)	106.1$	71.8	74.5	89.2	72.8	65.2
b	105.4	72.6	75.8	89.3	72.7	65.4
c (Cellulose Iβ centre)	104.3	71.8	75.4	88.4	71.3	66.0
**Tunicate ***						
a	106.1	71.8	74.5	89.2	72.9	65.2
c	104.4	71.8	75.3	88.4	71.4	66.0

† Iα and Iβ cellulose ^{13}C chemical shifts as given by Brouwer and Mikolajewski[23]
$ Same shifts for cellulose 1β given by Brouwer and Mikolajewski[23] except the C1 have been switched based on our assignments.
* ^{13}C chemical shift assignments acquired in this work for xylanase-treated hCNFs of poplar stem, with an error of ± 0.1 ppm.

SI Table 3 : Summary of Solid-state NMR experimental parameters								
Sample	Experiment	Recycle Delay (s)	Echo/ Mixing time (ms)	Spectral Width (F_2) (kHz)	Spectral Width (F_1) (kHz)	Acquisition time (t_2) (ms)	Acquisition time (t_1) (ms)	Number co-added FIDs
Poplar wood	1D CP	2	-	100		20	-	256
	1D DP	2	-	100		25	-	256
	1D DP	20	-	100		25	-	128
	2D CP refocussed INADEQUATE	2	2.24	59	37.5	20	6.67	128
	2D CP PDSD	2	30	75	37.5	25	8.2	64
	2D water edited CP refocussed INADEQUATE	2	^{1}H filter - 0.28<br>Diffusion time – 2<br>Mixing time – 2.24	28.5	100	20	4.7	270
Xylanase treated hCNFs of poplar wood	1D CP	2	-	100		20	-	256
	1D DP	2	-	100		25	-	256
	1D DP	20	-	100		25	-	128
	2D CP refocussed INADEQUATE	2	2.24	59	37.5	25	6.7	128
	2D CP PDSD	2	30	75	37.5	25	7.3	80
	2D CP PDSD	2	200	75	37.5	25	5.1	80
	2D CP PDSD	2	400	75	37.5	25	5.5	80
	2D Water edited CP PDSD	2	^{1}H filter - 0.32<br>Diffusion time – 2<br>Mixing time - 30	75	37.5	25	4.8	576
	2D water edited CP refocussed INADEQUATE	2	^{1}H filter - 0.28<br>Diffusion time – 2<br>Mixing time – 2.24	100	28.5	20	4.8	272

SI Table 4: Definitions of key terms used.

Key terms	Definition
tg/Domain 1 spectral region	Refers to the glucose environments of cellulose with ^{13}C NMR chemical shifts of ~89 ppm for C4 and ~65 ppm for C6 ppm.
gt and *gg*/ Domain 2 spectral region	Refers to the glucose environments of cellulose with ^{13}C NMR chemical shifts of ~84 ppm for C4 and ~62 ppm for C6 ppm.
Hydroxmethyl (*tg*) conformation ratio	The ratio of the area under the spectrum of the two spectral domains in the C4 region at ~89 ppm and ~84 ppm
Glucose environment	A glucose residue in cellulose in a particular local environment/ position in the microfibril such that it has distinct NMR chemical shifts.
Amorphous cellulose (in NMR terms)	Cellulose that has no long-range order and is seen on NMR spectrum as a very broad component.
Crystalline cellulose (in NMR terms)	Cellulose with long range order (at least over a 5 Å length scale) and has very narrow line shape in the NMR spectrum.
Local order	NMR is sensitive to the local environment of a nucleus; local order refers to the short-range order that NMR probes (up to 0.5 nm). The line widths of the glucan environments in cellulose are similar suggesting similar short-range order for all the environments assigned in this work. Diffraction techniques require long-range periodic order to get high resolution patterns.

SI Table 5: List of Acronyms

**Acronyms**	**Definition**
NMR	Nuclear Magnetic Resonance
MAS	Magic Angle Spinning
CP	Cross Polarisation
DP	Direct Polarisation
PDSD	Proton Driven Spin Diffusion
INADEQUATE	Incredible Natural Abundance Double Quantum Experiment
DQ	Double Quantum
SQ	Single Quantum
hCNFs	Holo-Cellulose Nanofibrils
D1	Spectral Domain 1
D2	Spectral Domain 2
TEM	Transmission Electron Microscopy
SCW	Secondary Cell Wall
